# MURC deficiency in smooth muscle attenuates pulmonary hypertension

**DOI:** 10.1038/ncomms12417

**Published:** 2016-08-22

**Authors:** Naohiko Nakanishi, Takehiro Ogata, Daisuke Naito, Kotaro Miyagawa, Takuya Taniguchi, Tetsuro Hamaoka, Naoki Maruyama, Takeru Kasahara, Masahiro Nishi, Satoaki Matoba, Tomomi Ueyama

**Affiliations:** 1 Department of Cardiovascular Medicine, Graduate School of Medical Science, Kyoto Prefectural University of Medicine, Kyoto 602-8566, Japan

## Abstract

Emerging evidence suggests that caveolin-1 (Cav1) is associated with pulmonary arterial hypertension. MURC (also called Cavin-4) is a member of the cavin family, which regulates caveolar formation and functions together with caveolins. Here, we show that hypoxia increased *Murc* mRNA expression in the mouse lung, and that *Murc*-null mice exhibited attenuation of hypoxia-induced pulmonary hypertension (PH) accompanied by reduced ROCK activity in the lung. Conditional knockout mice lacking *Murc* in smooth muscle also resist hypoxia-induced PH. MURC regulates the proliferation and migration of pulmonary artery smooth muscle cells (PASMCs) through Rho/ROCK signalling. Cav1 suppresses RhoA activity in PASMCs, which is reversed by MURC. MURC binds to Cav1 and inhibits the association of Cav1 with the active form of Gα13, resulting in the facilitated association of the active form of Gα13 with p115RhoGEF. These results reveal that MURC has a function in the development of PH through modulating Rho/ROCK signalling.

## Abstract (web summary)

MURC protein regulates the function of caveolae, the small invaginations of the plasma membrane in muscle cells. Here the authors show that by interacting with caveolin proteins, MURC affects RhoA/ROCK signalling and regulates proliferation and migration of pulmonary artery smooth muscle cells, suggesting a new target in therapy of pulmonary hypertension.

## Graphical abstract

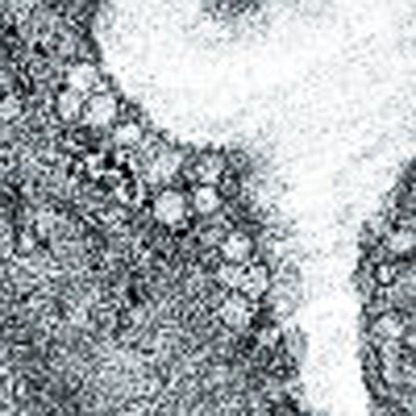

Pulmonary hypertension (PH) is a progressive disease of various origins, which results in right heart dysfunction and is associated with a poor prognosis^1^. Pulmonary arterial hypertension (PAH) is a clinical condition characterized by the presence of pre-capillary PH in the absence of other causes of pre-capillary PH such as PH due to lung diseases, chronic thromboembolism or other rare diseases^2^,^3^. PAH is subcategorized by its underlying causes, all of which are characterized by excessive pulmonary vasoconstriction and abnormal vascular remodelling processes^1^. Current treatments for PAH involve the use of prostanoids, endothelin receptor blockers and/or phosphodiesterase (PDE)-5 inhibitors^4^. Although these medications are effective in slowing the progression of PAH, they are insufficient to regress vascular remodelling. A clearer understanding of the mechanisms of vascular remodelling may lead to the development of novel therapeutic approaches for the prevention and/or treatment of PAH.

In heritable PAH, mutations in transforming growth factor-β/bone morphogenetic protein (TGF-β/BMP) receptors and activin receptor-like kinase type 1 (ALK-1) have been identified^1^,^5^. Recent research has shown that mutations in caveolin-1 (Cav1), a major component of caveolae, are also associated with PAH^3^,^6^. Caveolae are involved in several important cellular processes, including signal transduction, endocytosis and cholesterol homeostasis, and caveolins serve as the essential structural components of caveolae and function as scaffolds for caveolar-mediated signalling pathways^7^,^8^,^9^. Cav1 is the predominant caveolin isoform in smooth muscle cells (SMCs), and Cav1 deficiency results in the loss of caveolae in SMCs^10^,^11^. *Cav1*-null (*Cav1*^−/−^) mice develop PH and right ventricular (RV) hypertrophy associated with pulmonary vascular remodelling^12^,^13^.

Increasing evidence suggests that the Rho/ROCK pathway is activated in PH, and that the inhibition of ROCK activity attenuates PH^14^,^15^,^16^,^17^,^18^,^19^. We have identified MURC (muscle-restricted coiled-coil protein), also referred to as Cavin-4, and reported that it is expressed in vascular SMCs (VSMCs), cardiomyocytes, and skeletal muscle cells, and regulates the Rho/ROCK pathway in cardiomyocytes^20^,^21^,^22^,^23^,^24^. In the cavin family, polymerase I and transcript release factor (PTRF, also referred to as Cavin-1), serum deprivation protein response (SDPR, also referred to as Cavin-2), and SDR-related gene product that binds to C kinase (SRBC, also referred to as Cavin-3) have been associated with Cav1 and shown to be crucial for caveolar formation or morphology^23^,^25^,^26^,^27^,^28^,^29^,^30^. In patients with *PTRF* mutations and *Ptrf*-null (*Ptrf*^−/−^) mice, caveolae disappear and the protein expression of caveolins is markedly reduced^22^,^27^,^29^. In addition, *Ptrf*^−/−^ mice have been shown to exhibit elevated pulmonary arterial pressure^31^. However, the roles of MURC in SMCs and in the development of PH remain unknown.

Here, we show that MURC is associated with Cav1 and caveolin-3 (Cav3), and that MURC in smooth muscle is implicated in the development of PH. The association of MURC with Cav1 regulates RhoA signalling in VSMCs.

## Results

### Colocalization of MURC with Cav1 and Cav3

**Figure 1:**
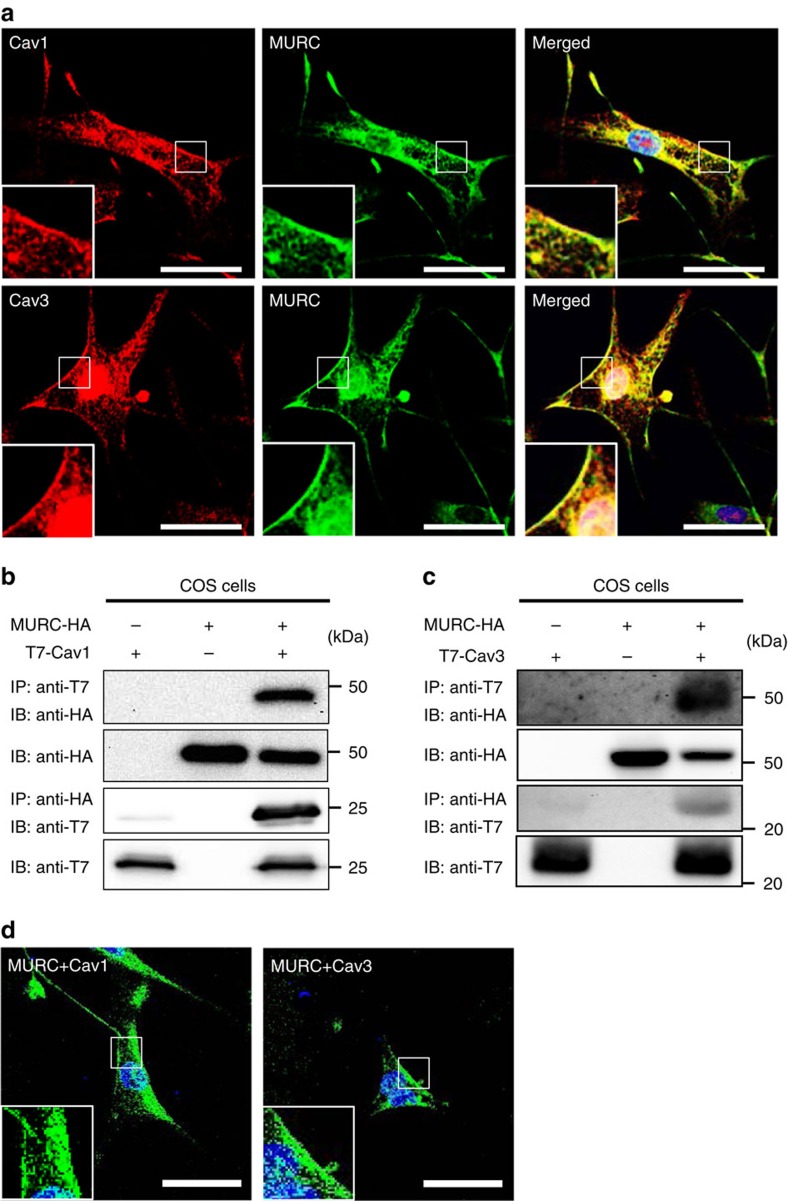
Colocalization of MURC with Cav1 and Cav3 in hPASMCs. (**a**) Representative immunostaining images of MURC, Cav1 and Cav3 in hPASMCs. Scale bar, 50 μm. (**b**,**c**) COS cells were transfected with pcDNA3.1-hMURC-HA and/or pcDNA3.1-T7-hCav1 (**b**) or pcDNA3.1-T7-hCav3 (**c**), and the cell lysates were immunoprecipitated with anti-HA and anti-T7 antibodies. (**d**) The *in situ* association of proteins in hPASMCs was assessed by the BiFC assay, which detects fluorescent signals in living cells when proteins associate with each other. Upper, hPASMCs were transfected with phmKGC-MC-hMURC and phmKGN-MC-hCav1. Lower, hPASMCs were transfected with phmKGN-MN-hMURC and phmKGC-MN-hCav3. Scale bar, 50 μm. Uncropped images of blots are shown in Supplementary Fig. 6.

Cav1 and Cav3 are expressed in VSMCs^11^,^32^. To examine the subcellular localization of MURC, Cav1 and Cav3 in PASMCs, we performed immunostaining using human PASMCs (hPASMCs) with anti-MURC, anti-Cav1 and anti-Cav3 antibodies. MURC was colocalized with Cav1 and Cav3 at the plasma membrane of hPASMCs (Fig. 1a). Immunoprecipitation showed the association of MURC with Cav1 and Cav3 (Fig. 1b,c). The bimolecular fluorescent complementation (BiFC) assay in hPASMCs also revealed the association of MURC with Cav1 and Cav3 *in situ*, which was mainly detected at the plasma membrane (Fig. 1d).

### Attenuation of hypoxia-induced PH in *Murc*-knockout mice

**Figure 2:**
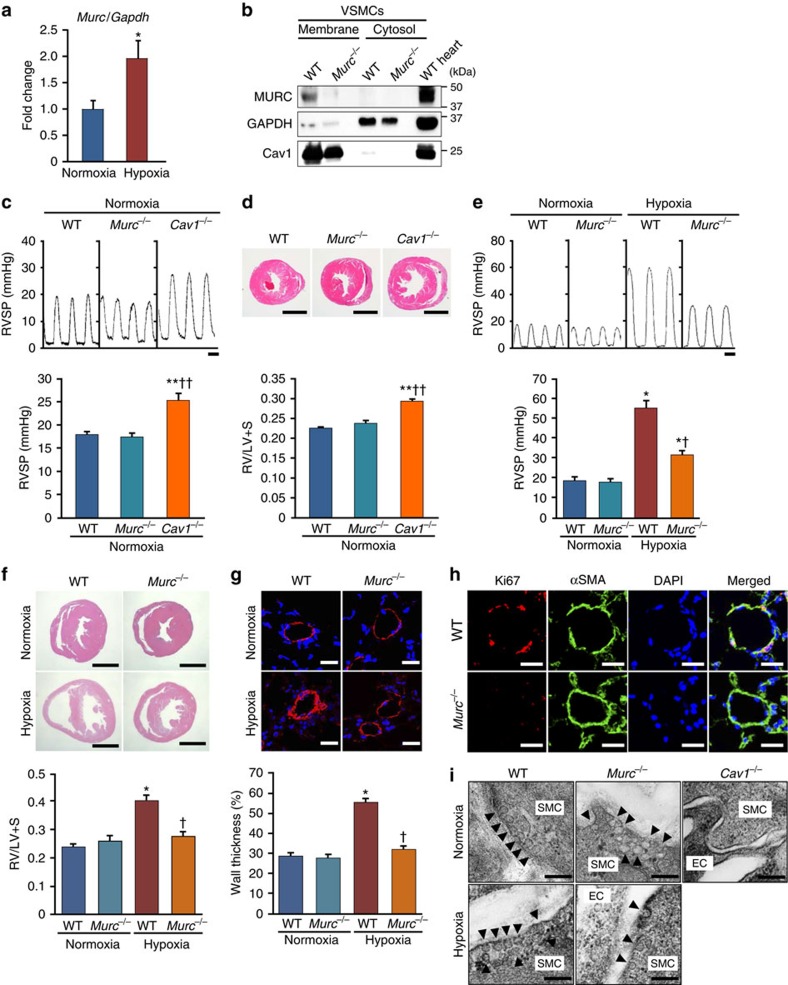
Attenuation of PH induced by hypoxia in *Murc*^−/−^ mice. (**a**) Expression of Murc mRNA in the lung exposed to normoxia or hypoxia for 6 weeks (*n*=6 per group). *P<0.05 compared with respective normoxic group. (**b**) VSMCs were isolated from WT and *Murc*^−/−^ aortae. Lysates of WT and *Murc*^−/−^ VSMCs, and WT hearts were immunoblotted with an anti-MURC antibody. Membrane and cytosol fractions were separated by the gradient of sucrose. (**c**) RV systolic pressure (RVSP) in WT, *Murc*^−/−^ and *Cav1*^−/−^ mice at the age of 16 weeks under normoxic conditions. To measure RV hemodynamics, open-chest RV catheterization using a 1.2-F pressure catheter was performed during anaesthesia with 1.5% isoflurane (*n*=4–6 per group). Scale bar, 100 ms. **P<0.01 compared with WT mice, ^††^*P*<0.01 compared with *Murc*^−/−^ mice. (**d**) Relative RV weight was determined as the ratio of the RV weight to LV and septum weights (RV/LV+S) (*n*=4–10 per group). Scale bar, 2 mm. **P<0.01 compared with WT mice, ^††^*P*<0.01 compared with *Murc*^−/−^ mice. (**e**) RVSP in WT and *Murc*^−/−^ mice under hypoxic conditions. WT and *Murc*^−/−^ mice were exposed to normobaric hypoxia (10% O_2_) for 4 weeks (*n*=6–11 per group). Scale bar, 100 ms. *P<0.05 compared with the respective normoxic group, ^†^*P*<0.05 compared with the hypoxic WT group. (**f**) Relative RV weight was determined as RV/LV+S (*n*=4–7 per group). Scale bar, 2 mm. *P<0.05 compared with the normoxic WT group, ^†^*P*<0.05 compared with the hypoxic WT group. (**g**) Pulmonary vascular remodelling was assessed by measuring the medial thickness of alveolar/distal pulmonary vessels of 25–100 μm in diameter from lung sections immunostained with αSMA from 5–6 images from different fields (*n*=4–5 per group). Per cent wall thickness is expressed as the medial wall area divided by the area of the vessel. Scale bar, 20 μm. *P<0.05 compared with the normoxic WT group, ^†^*P*<0.05 compared with the hypoxic WT group. (**h**) The proliferation of VSMCs was evaluated by Ki67 staining in the hypoxia-exposed lung for 2 weeks. Scale bar, 20 μm. (**i**) Caveolae of the pulmonary arterial SMCs were observed by electron microscopy. Scale bar, 200 nm. Data are presented as mean±s.e.m.

No animal model completely recapitulates human PAH^4^. However, since chronic normobaric hypoxia is commonly used as a model of PH in mice^33^, we employed this animal model in the present study. We exposed wild-type (WT) mice to normobaric hypoxia and examined the expression level of *Murc* mRNA in the lung. *Murc* mRNA expression levels were higher in the lung exposed to hypoxia than in that under normoxia (Fig. 2a). This finding raises the possibility that Murc has a role in the development of PH. Therefore, we examined this hypothesis using *Murc*-knockout (*Murc*^−/−^) mice^34^. We firstly examined the protein expression of Murc in WT and *Murc*^−/−^ VSMCs. Most of the Murc protein was detected in the membrane fraction of VSMCs isolated from WT mice, while it was undetectable in VSMCs isolated from *Murc*^−/−^ mice (Fig. 2b), confirming the deletion of Murc in VSMCs. Cav1 expression was observed in the membrane fraction of both WT and *Murc*^−/−^ VSMCs. Moreover, Cav1 protein expression in *Murc*^−/−^ lungs was not significantly different from that in WT lungs (Supplementary Fig. 1a).

Cav1 deficiency has been shown to cause hypertensive pulmonary phenotypes^12^,^13^. We compared RV systolic pressure (RVSP) and the ratio of the RV to left ventricle (LV) and septum weights (RV/LV+S) among WT, *Murc*^−/−^ and *Cav1*^−/−^ mice. Under normoxic conditions, no significant differences were observed in systolic blood pressure, diastolic BP, heart rate, left ventricular systolic function, RVSP, or RV/LV+S between WT and *Murc*^−/−^ mice, while *Cav1*^−/−^ mice showed significant elevations in RVSP with RV hypertrophy, as assessed by RV/LV+S (Supplementary Table 1; Fig. 2c,d).

To explore the functional significance of Murc in the development of PH, WT and *Murc*^−/−^ mice were exposed to normobaric hypoxia for 4 weeks. After hypoxia, WT mice displayed significant elevations in RVSP with RV hypertrophy, whereas *Murc*^−/−^ mice showed the attenuation of RVSP elevations and RV hypertrophy (Fig. 2e,f). Vascular remodelling, which is characterized by an increase in the wall thickness of pulmonary arterioles, as evaluated by the α-smooth muscle actin (αSMA)-positive area, was observed in WT mice after hypoxia, while it was significantly attenuated in *Murc*^−/−^ mice (Fig. 2g), suggesting that VSMC proliferation is suppressed in the latter group. In accordance with these findings, PASMC proliferation evaluated by Ki67 staining was suppressed in the pulmonary vessels of *Murc*^−/−^ mice exposed to hypoxia for 2 weeks (Fig. 2h). Caveolae in PASMCs were retained in *Murc*^−/−^ mice, and hypoxia did not affect the caveolar morphology in WT or *Murc*^−/−^ mice, while caveolae disappeared in the lung of *Cav1*^−/−^ mice (Fig. 2i).

**Figure 3:**
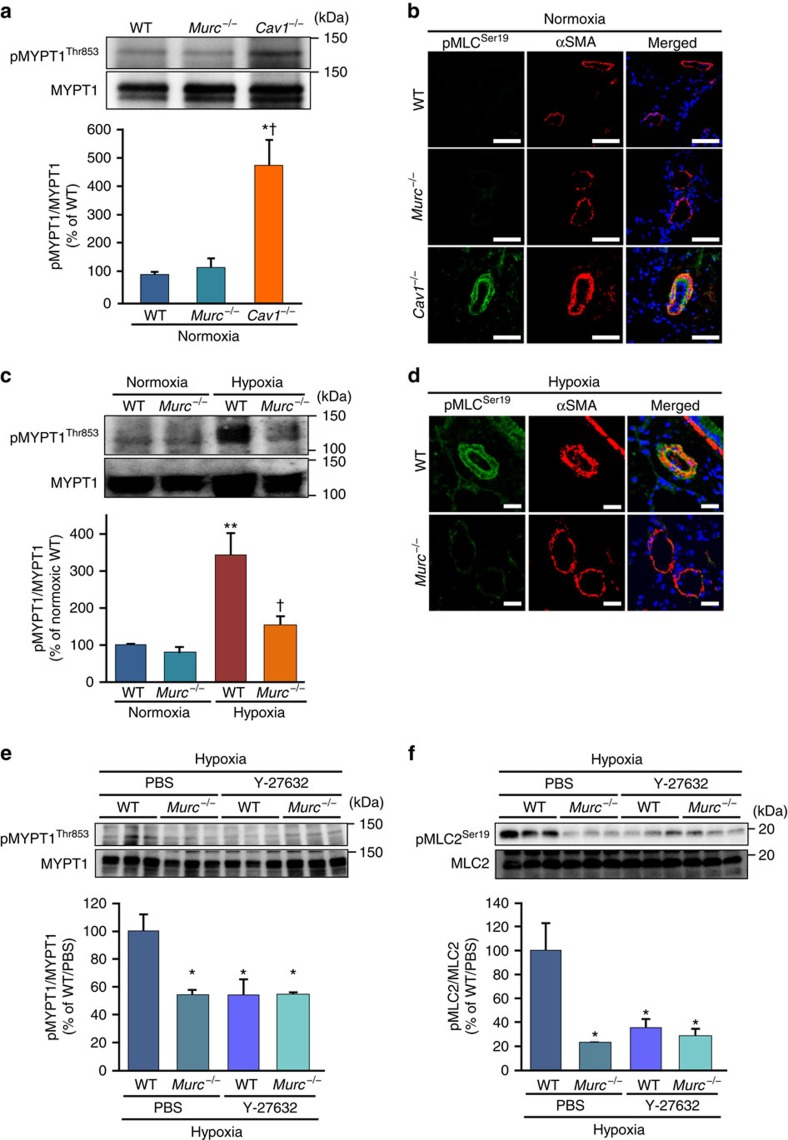
Attenuation of MYPT1 and MLC2 phosphorylation in the *Murc*^−/−^ lung. (**a**) MYPT1 phosphorylation in WT, *Murc*^−/−^ and *Cav1*^−/−^ mice at the age of 16 weeks under normoxic conditions (*n*=3 per group). *P<0.05 compared with WT mice, ^†^*P*<0.05 compared with *Murc*^−/−^ mice. (**b**) The phosphorylation of MLC2 was assessed in the lungs of WT, *Murc*^−/−^, and *Cav1*^−/−^ mice at the age of 16 weeks under normoxic conditions. Lung sections were immunostained with pMLC2^Ser19^ and αSMA. Scale bar, 50 μm. (**c**) MYPT1 phosphorylation in WT and *Murc*^−/−^ mice under hypoxic conditions (*n*=3 per group). **P<0.01 compared with normoxic WT group, ^†^*P*<0.05 compared with hypoxic WT group. (**d**) The phosphorylation of MLC2 was assessed in the lung exposed to hypoxia for 4 weeks. Lung sections were immunostained with pMLC2^Ser19^ and αSMA. Scale bar, 20 μm. (**e**) MYPT1 phosphorylation in the lungs of WT and *Murc*^−/−^ mice treated with either PBS or Y-27632 (30 mg kg^−1^·day^−1^ for 4 weeks) under hypoxic conditions (*n*=4–6 per group). *P<0.05 compared with the PBS-treated WT group exposed to hypoxia. (**f**) MLC2 phosphorylation in the lungs of WT and *Murc*^−/−^ mice treated with either PBS or Y-27632 (30 mg kg^−1^·day^−1^ for 4 weeks) under hypoxic conditions (*n*=3 per group). *P<0.05 compared with the PBS-treated WT group exposed to hypoxia. Data are presented as mean±s.e.m. Uncropped images of blots are shown in Supplementary Fig. 6.

We previously showed that Murc regulates Rho/ROCK signalling in cardiomyocytes^20^. The Rho/ROCK signalling pathway modulates the ‘Ca^2+^ sensitivity' of smooth muscle mainly by suppressing myosin light chain (MLC) phosphatase activity, thereby regulating vascular smooth muscle tone^35^; therefore, we evaluated the activity of ROCK to examine its downstream targets, myosin phosphatase targeting subunit 1 (MYPT1) of MLC phosphatase and MLC2 (refs 36, 37) in the lung of WT and *Murc*^−/−^ mice. ROCK has been shown to phosphorylate MYPT1 at Thr853 and Thr696, which results in a decrease in MLC phosphatase activity and increase in MLC phosphorylation^35^. ROCK also phosphorylates MLC at Ser19 to increase myosin ATPase activity^38^. Under normoxic conditions, the phosphorylation of MYPT1 at Thr853, which is considered to be a specific target of ROCK^36^,^39^, did not significantly differ between WT and *Murc*^−/−^ lungs, while the phosphorylation of MYPT1 at Thr853 was greater in *Cav1*^−/−^ lungs than in WT and *Murc*^−/−^ lungs (Fig. 3a). In addition, the greater phosphorylation of MLC2 at Ser19 in the PASMC of *Cav1*^−/−^ mice was observed by immunostaining (Fig. 3b). After hypoxia, MYPT1 phosphorylation at Thr853 was significantly greater in the lung of WT mice than in the lung of *Murc*^−/−^ mice (Fig. 3c). These findings suggest that hypoxia-induced ROCK activation in the lung is reduced by Murc deficiency, which is supported by the immunostaining analysis showing that the phosphorylation of MLC2 at Ser19 was less in the PASMCs of *Murc*^−/−^ mice exposed to hypoxia than in those of WT mice exposed to hypoxia (Fig. 3d). Furthermore, a treatment with Y-27632, a selective ROCK inhibitor, inhibited the phosphorylation of MYPT1 and MLC2 in the lung of WT mice exposed to hypoxia, but not in the lung of *Murc*^−/−^ mice exposed to hypoxia (Fig. 3e,f).

**Figure 4:**
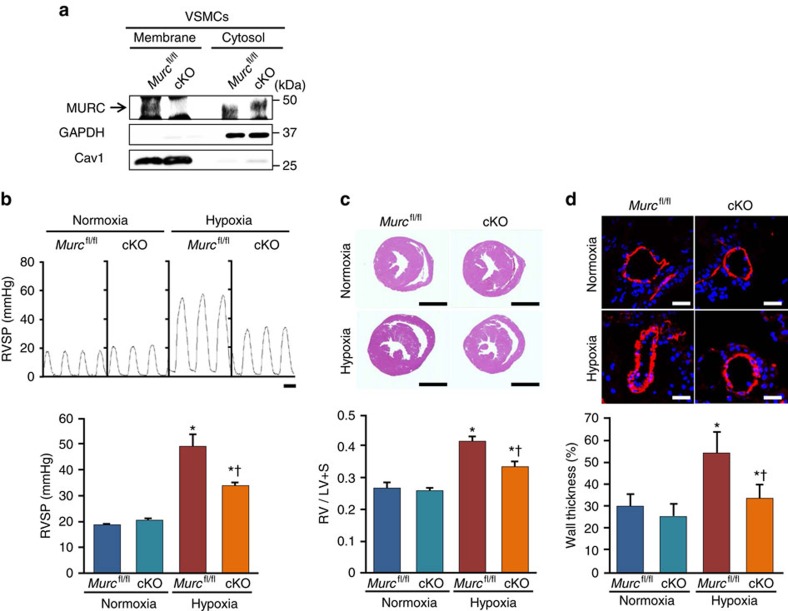
Attenuation of PH induced by hypoxia in *Murc* conditional knockout mice. (**a**) VSMCs were isolated from *Murc*^*fl/fl*^ and cKO aortae. Lysates of *Murc*^*fl/fl*^ and cKO VSMCs were immunoblotted with an anti-MURC antibody. Membrane and cytosol fractions were separated by the gradient of sucrose. (**b**) *Murc*^*fl/fl*^ and cKO mice were exposed to normobaric hypoxia (10% O_2_) for 4 weeks, and RV hemodynamics was then measured (*n*=5–7 per group). Scale bar, 100 ms. (**c**) Relative RV weights of *Murc*^*fl/fl*^ and cKO mice were determined as the ratio of the RV weight to the LV and septum weights (RV/LV+S) (*n*=6–7 per group). Scale bar, 2 mm. (**d**) Pulmonary vascular remodelling was assessed by measuring the medial thickness of alveolar/distal pulmonary vessels of 25–100 μm in diameter from lung sections immunostained with αSMA from 5–6 images from different fields (*n*=4–5 per group). Per cent wall thickness is expressed as the medial wall area divided by the area of the vessel. Scale bar, 20 μm. *P<0.05 compared with the respective normoxic group, ^†^*P*<0.05 compared with the hypoxic WT group. Data are presented as mean±s.e.m. Uncropped images of blots are shown in Supplementary Fig. 6.

To further examine the role of Murc in vascular smooth muscle in the development of PH and RV remodelling, we generated *Murc* conditional knockout (cKO, *SM22Cre*^*+*^*Murc*^*fl/fl*^) mice by mating *SM22Cre* transgenic mice with *Murc*^*fl/fl*^ mice. In cKO mice, the Murc protein was deleted in VSMCs, but was retained in the skeletal muscle (Fig. 4a; Supplementary Fig. 1b). The Murc protein was detected in the cKO heart by western blotting, but was significantly decreased (Supplementary Fig. 1b). Murc mRNA and protein were detected by RT-quantitative PCR and immunostaining, respectively (Supplementary Fig. 1c,d). In addition, we performed hematoxylin and eosin (H&E) staining on the aorta of WT, *Murc*^−/−^, *Murc*^fl/fl^ and cKO mice. H&E staining revealed no significant differences in vascular walls among WT, *Murc*^−/−^, *Murc*^fl/fl^ and cKO mice (Supplementary Fig. 1e).

After exposure to normobaric hypoxia for 4 weeks, hypoxia-induced RVSP elevations, RV hypertrophy and vascular remodelling were less in cKO mice than in *Murc*^*fl/fl*^ mice (Fig. 4b–d). In addition, we performed morphometric and echocardiographic analyses to assess cardiac function in *Murc*^fl/fl^ and cKO mice. Under normoxia, no significant differences were noted in morphometric or echocardiographic parameters between *Murc*^fl/fl^ and cKO mice (Supplementary Table 2). Furthermore, after exposure to normobaric hypoxia for 4 weeks, LV systolic function in cKO mice was not significantly different from that in *Murc*^fl/fl^ mice (Supplementary Table 3). These results suggest that LV function does not affect pulmonary arterial pressure in cKO mice under hypoxia, and that Murc in PASMCs plays crucial roles in the development of PH and RV remodelling.

### MURC regulates p115RhoGEF/Rho/ROCK signalling in PASMCs

Hypoxia and hypoxia-associated stimuli induce the production of various chemokines/cytokines and growth factors in the pulmonary artery^4^. Among them, TGF-β1 mRNA and protein expression is induced by hypoxia in PASMCs and the lung^40^,^41^, and its signalling has been shown to mediate hypoxia-induced pulmonary arterial remodelling^42^. Since *MURC* mRNA expression in the lung is induced by hypoxia, we determined whether TGF-β1, IL-1β and endothelin-1 (ET-1) induce *MURC* mRNA expression in hPASMCs. TGF-β1 induced *MURC* mRNA expression in hPASMCs, whereas IL-1β and ET-1 did not (Supplementary Fig. 2), suggesting that TGF-β1 is one of the upstream regulators involved in hypoxia-induced MURC expression in PASMCs.

**Figure 5:**
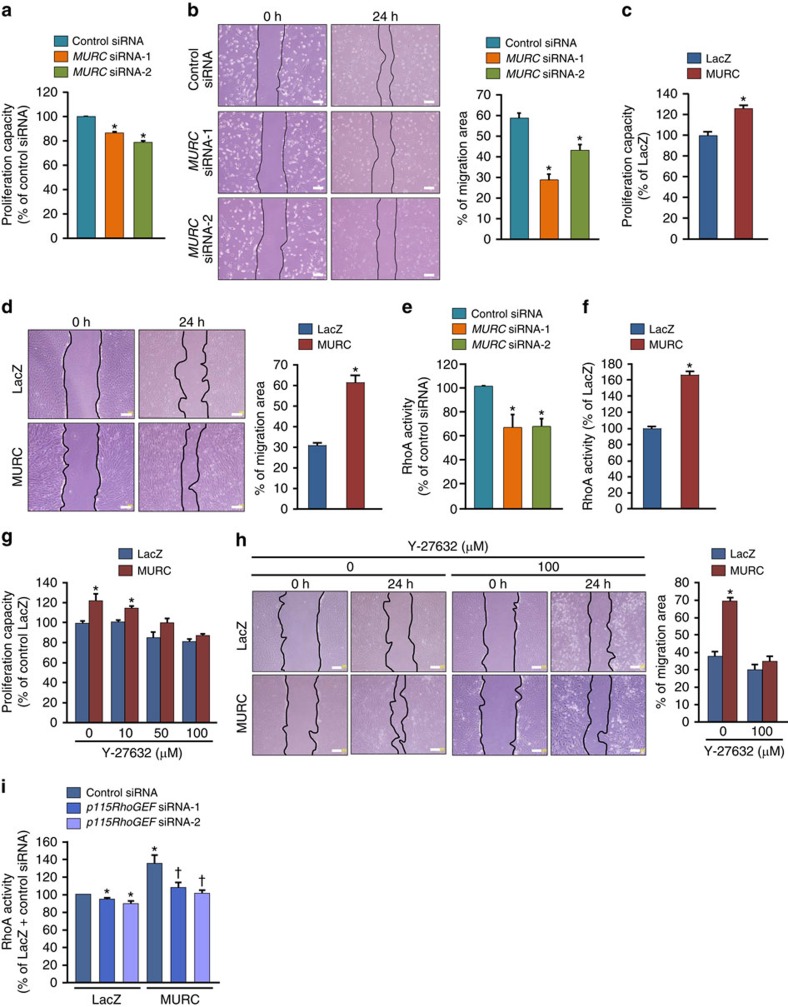
MURC regulates hPASMC proliferation and migration. (**a**) The proliferation capacity in hPASMCs transfected with control siRNA or *MURC* siRNA was assessed using a WST-1 cell proliferation assay system (*n*=10 per group). *P<0.05 compared with control siRNA. (**b**) Migration in hPASMCs transfected with control siRNA or *MURC* siRNAs was assessed by a wound healing assay (*n*=8 per group). Wound closure was quantified by the per cent change in the wound area. Scale bar, 200 μm. *P<0.05 compared with control siRNA. (**c**,**d**) Proliferation (*n*=8 per group) and migration (*n*=7–8 per group) capacities were assessed in MURC-overexpressing hPASMCs. Scale bar, 200 μm. *P<0.05 compared with LacZ. (**e**) RhoA activity was measured in hPASMCs transfected with control siRNA or *MURC* siRNAs (*n*=6 per group). Starved cells were stimulated with 1% FBS for 1 h. *P<0.05 compared with control siRNA. (**f**) RhoA activity was measured in LacZ- and MURC-overexpressing hPASMCs (*n*=3 per group). *P<0.05 compared with LacZ. Proliferation (**g**) and migration (**h**) capacities were assessed in hPASMCs incubated with or without a ROCK inhibitor, Y-27632 (*n*=3–5 per group). Scale bar, 200 μm. (**i**) RhoA activity was assessed in hPASMCs transduced with LacZ and MURC with control siRNA or *p115RhoGEF* siRNAs (*n*=8 per group). hPASMCs were infected with a puromycin-resistant retrovirus expressing LacZ and MURC-FLAG. After selection using puromycin, hPASMCs were transfected with control siRNA, p115RhoGEF siRNA-1, or p115RhoGEF siRNA-2. *P<0.05 compared with LacZ+control siRNA, ^†^*P*<0.05 compared with MURC+control siRNA. Data are presented as mean±s.e.m.

To elucidate the mechanisms by which MURC modulates pulmonary vascular remodelling, we knocked down MURC expression in hPASMCs using human *MURC*-specific siRNA (Supplementary Fig. 3a) and performed proliferation and migration assays using MURC-knockdown hPASMCs. Fetal bovine serum (FBS)-induced proliferation in MURC-knockdown hPASMCs was less than that in hPASMCs transfected with control siRNA (Fig. 5a). Wound healing and Boyden chamber assays revealed that migration was also less in MURC-knockdown hPASMCs than in control hPASMCs (Fig. 5b; Supplementary Fig. 3b). We then established MURC-overexpressing hPASMCs using a recombinant retrovirus expressing MURC (Supplementary Fig. 3c). hPASMCs overexpressing MURC showed increase in proliferation and migration (Fig. 5c,d). In accordance with these findings, FBS-induced proliferation and migration in VSMCs isolated from *Murc*^−/−^ mice were significantly less than those in WT VSMCs (Supplementary Fig. 3d,e).

In SMCs, the Rho/ROCK pathway has been demonstrated to regulate proliferation and migration^43^,^44^. We examined whether MURC regulates the proliferation and migration of PASMCs through the Rho/ROCK pathway. RhoA activity in MURC-knockdown hPASMCs was significantly weaker than that in control hPASMCs (Fig. 5e). Correspondingly, RhoA activity was weaker in *Murc*^−/−^ VSMCs than in WT VSMCs (Supplementary Fig. 3f). MURC overexpression increased RhoA activity in hPASMCs (Fig. 5f). Furthermore, MURC knockdown attenuated FBS-induced MYPT1 and MLC2 phosphorylation in hPASMCs, while MURC overexpression induced MYPT1 and MLC2 phosphorylation in hPASMCs (Supplementary Fig. 3g,h). We then used Y-27632 to assess the involvement of Rho/ROCK signalling in the MURC-induced proliferation and migration of hPASMCs. Y-27632 suppressed the MURC-induced proliferation of hPASMCs in a dose-dependent manner (Fig. 5g). Y-27632 also inhibited the MURC-induced migration of hPASMCs (Fig. 5h). Moreover, hydroxyfasudil, another ROCK inhibitor, suppressed MURC-induced proliferation and migration in rat VSMCs (Supplementary Fig. 3i,j). Collectively, these results indicate that MURC-induced proliferation and migration are mediated by the Rho/ROCK pathway in hPASMCs.

Guanine nucleotide exchange factors (GEFs) stimulate the exchange of GDP for GTP to generate the activated form of Rho proteins^43^,^45^. Among RhoGEFs, p115RhoGEF (also referred to as Arhgef1) and leukemia-associated Rho guanine nucleotide exchange factor (LARG) have been shown to regulate vascular tone^46^,^47^. To examine the functional significance of p115RhoGEF in MURC-induced RhoA activation, we used human *p115RhoGEF*-specific siRNA. The knockdown of p115RhoGEF suppressed MURC-induced RhoA activation in hPASMCs (Fig. 5i). We also used a mutant of p115RhoGEF [p115(2A)] that contains alanine point substitutions of two residues (E423 and N603) in the Dbl homology (DH) domain, which is responsible for the catalytic exchange reaction. RhoA activity in hPASMCs was reduced more by p115(2A) overexpression than by LacZ overexpression, indicating that p115(2A) acts in a dominant negative fashion. MURC-induced RhoA activation was significantly attenuated by p115(2A) (Supplementary Fig. 3k). Collectively, these findings indicate that p115RhoGEF mediates MURC-induced RhoA activation.

### Dissociation between Cav1 and activated Gα13 by MURC

**Figure 6:**
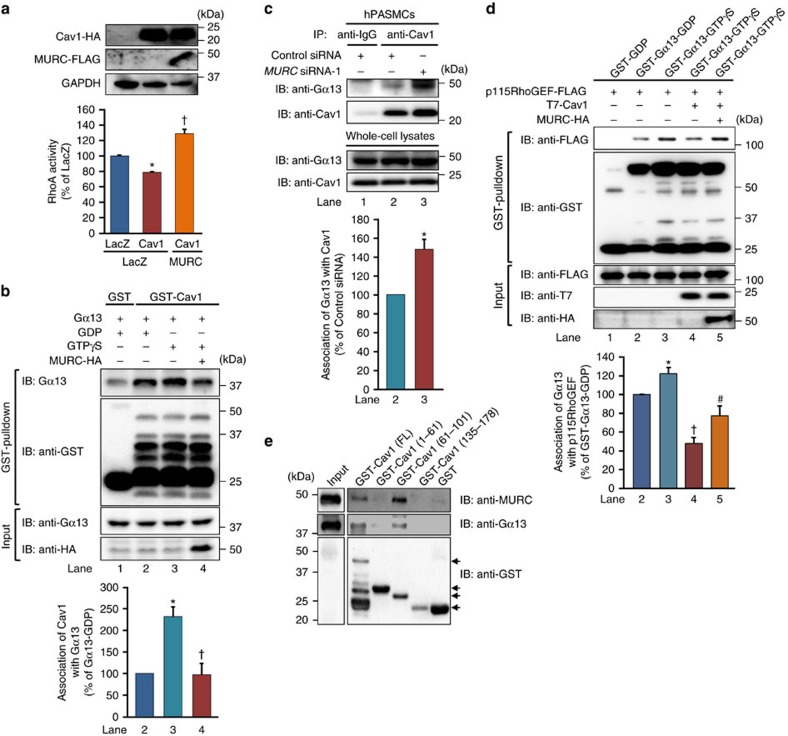
Inhibition of the association of Cav1 with Gα13 by MURC. (**a**) RhoA activity was assessed in hPASMCs transduced with LacZ, Cav1-HA and Cav1-HA and MURC-FLAG (*n*=4 per group). hPASMCs were infected with a hygromycin-resistant retrovirus expressing LacZ and Cav1-HA. After selection using hygromycin, hPASMCs were infected with a puromycin-resistant retrovirus expressing LacZ and MURC-FLAG, and subsequently selected using puromycin. *P<0.05 compared with LacZ+LacZ, ^†^*P*<0.05 compared with LacZ+Cav1. (**b**) COS cells were transfected with pcDNA3.1-hMURC-HA and/or pcDNA3.1-hGα13. The COS cell lysates incubated with GDP or GTPγS and GST pulldown was performed with GST-fusion Cav1 conjugated to glutathione-Sepharose beads. Precipitated proteins were blotted with anti-Gα13 and anti-GST antibodies (*n*=3 per group). *P<0.05 compared with Gα13-GDP+Cav1, ^†^*P*<0.05 compared with Gα13-GTPγS+Cav1. (**c**) The association of Gα13 with Cav1 was assessed in hPASMCs transfected with control siRNA or *MURC* siRNA (*n*=3 per group). *P<0.05 compared with control siRNA. (**d**) GST-fusion Gα13 conjugated to glutathione-Sepharose beads was preloaded with GDP or GTPγS. GST pulldown was performed with COS cell lysates transfected with plasmids expressing the indicated proteins. Precipitated proteins were blotted with anti-FLAG and anti-GST antibodies (*n*=5 per group). *P<0.05 compared with Gα13-GDP+p115RhoGEF, ^†^*P*<0.05 compared with Gα13-GTPγS+p115RhoGEF, ^#^*P*<0.05 compared with Gα13-GTPγS+p115RhoGEF+Cav1. (**e**) Each domain of Cav1 (FL: full length, 1–61: C-terminal domain, 61–101: oligomerization domain, 135–178: N-terminal domain) tagged with GST was conjugated to glutathione-Sepharose beads, and incubated with lysates from COS cells transfected with pcDNA3.1-hMURC-FLAG or pcDNA3.1-hGα13. Pulled-down protein was blotted with anti-MURC or anti-Gα13 antibodies. Arrows indicate each GST-fusion protein. Data are presented as mean±s.e.m. Uncropped images of blots are shown in Supplementary Fig. 6.

G-protein-coupled receptors (GPCRs) for various vasoconstrictors, such as ET-1 and thrombin, couple to Gα12/13, and activated Gα13 binds to p115RhoGEF, which enhances the activity of p115RhoGEF, leading to Rho activation^45^,^48^,^49^. The absence of Cav1 has been shown to increase RhoA activity in VSMCs^11^. To examine the relationship between Cav1 and MURC and its effects on RhoA activity in hPASMCs, Cav1 and MURC were transduced with recombinant retroviruses expressing Cav1 and MURC into hPASMCs. Cav1 overexpression suppressed RhoA activity in hPASMCs, which was reversed by MURC overexpression (Fig. 6a), suggesting that Cav1 negatively regulates RhoA activity, and that MURC counteracts the effects of Cav1 on the activation of RhoA. Cav1 has been identified as a potent inhibitor of heteromeric G-proteins^9^. Therefore, we assumed that Cav1 inhibits RhoA activation through its association with Gα13, which is modulated by MURC. To test this, we performed glutathione S-transferase (GST)-pulldown assays using GST-Cav1 and GST-Gα13. Cav1 exhibited higher affinity for the GTPγS (a non-hydrolyzable GTP analogue)-bound form of Gα13 (Gα13-GTPγS) than for the GDP-bound form of Gα13 (Gα13-GDP), and MURC reduced the association of Cav1 with Gα13-GTPγS (Fig. 6b). Cav1 also had higher affinity for a constitutively active mutant of Gα13, Gα13(Q226L), than for Gα13 (Supplementary Fig. 4a). In addition, Cav1 had higher affinity for Gα13 incubated with GTPγS than for Gα13 incubated with GDP (Supplementary Fig. 4b). These findings suggest the preferential binding of Cav1 to active Gα13 over inactive Gα13. Furthermore, immunoprecipitation revealed that Cav1-associated Gα13 was more prominent in MURC-knockdown hPASMCs than in hPASMCs transfected with control siRNA (Fig. 6c).

We next examined the association of Gα13 with p115RhoGEF. In the GST-pulldown assay using GST-Gα13, Gα13-GTPγS had higher affinity for p115RhoGEF than Gα13-GDP, and Cav1 reduced the association of p115RhoGEF with Gα13-GTPγS, which was reversed by MURC (Fig. 6d). Immunoprecipitation also demonstrated that p115RhoGEF had higher affinity for Gα13 incubated with GTPγS than for Gα13 incubated with GDP, and that Cav1 reduced the association of p115RhoGEF with Gα13 incubated with GTPγS, which was reversed by MURC (Supplementary Fig. 4c). We then attempted to identify the domain of Cav1 responsible for the associations of both Gα13 and MURC. Cav1(61–101), which is the oligomerization domain containing a scaffolding domain in the distal half, associated with MURC and Gα13 (Fig. 6e). These findings suggest that MURC and the active form of Gα13 are competitively associated with Cav1, and that the association of MURC with Cav1 decreases that between Cav1 and the active form of Gα13, thereby facilitating the active form of Gα13 to interact with p115RhoGEF.

### Association of MURC with p115RhoGEF and RhoA

**Figure 7:**
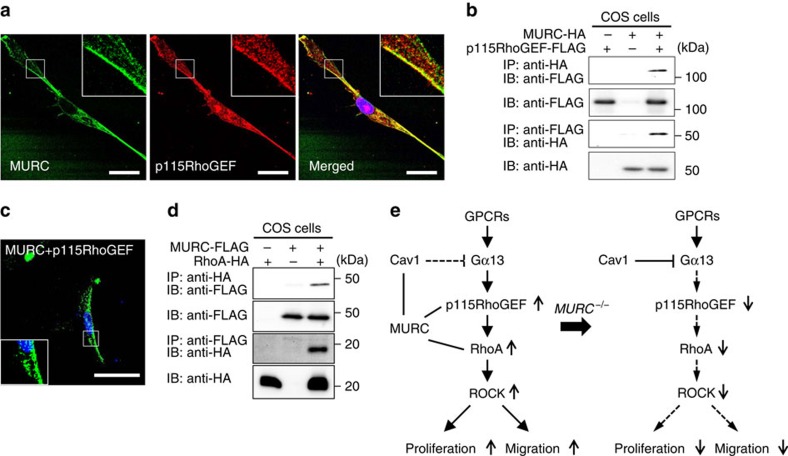
Association of MURC with RhoA and p115RhoGEF. (**a**) Immunostaining images of MURC and p115RhoGEF in hPASMCs. Scale bar, 20 μm. (**b**) pcDNA3.1-hMURC-HA and/or pCS2FLAG-hp115RhoGEF were cotransfected into COS cells, and the cell lysates were then immunoprecipitated with anti-HA and anti-FLAG antibodies. (**c**) hPASMCs were transfected with phmKGN-MC-hMURC and phmKGC-MN-hp115RhoGEF. Scale bar, 50 μm. (**d**) COS cells were transfected with pcDNA3.1-hMURC-FLAG and/or pcDNA3.1-hRhoA-HA and cell lysates were immunoprecipitated with anti-FLAG and anti-HA antibodies. (**e**) Proposed roles of the MURC-mediated activation of Rho/ROCK signalling in PASMCs. When MURC is present, it associates with Cav1, leading to reductions in the association between Cav1 and Gα13, which facilitates the interaction of Gα13 with p115RhoGEF, thereby activating the Rho/ROCK pathway. MURC also serves as a platform to compartmentalize p115RhoGEF and RhoA at caveolae. In the absence of MURC, Cav1 interacts with Gα13 and inhibits the association of Gα13 with p115RhoGEF, leading to the suppression of the Rho/ROCK pathway. Uncropped images of blots are shown in Supplementary Fig. 6.

Since caveolae have been recognized to serve as signalling platforms for complexes of receptors, signal components and their targets^8^,^9^,^50^, we investigated the localization of MURC and p115RhoGEF. Immunostaining showed that MURC was colocalized with p115RhoGEF in hPASMCs (Fig. 7a). MURC was immunoprecipitated with p115RhoGEF, and the BiFC assay revealed the association of MURC with p115RhoGEF in hPASMCs (Fig. 7b,c). The association of MURC with p115RhoGEF was not inhibited by Cav1 (Supplementary Fig. 5). Furthermore, MURC was immunoprecipitated with RhoA (Fig. 7d). Thus, MURC has the ability to induce clustering molecules involved in RhoA signalling.

## Discussion

In SMCs, Cav1 is predominantly expressed among caveolins and is the predominant determinant of caveolar morphology^10^,^11^. We showed here that MURC is primarily distributed at the plasma membrane of PASMCs, in which MURC is associated with Cav1 and Cav3. Even in *Murc*^−/−^ VSMCs, Cav1 is expressed and located at the plasma membrane, and caveolae are retained. These observations indicate that although MURC is located together with Cav1 and Cav3 at the caveolae of VSMCs, MURC is not essential for the formation of caveolae in VSMCs. However, our observations also indicate that MURC regulates caveolar-mediated RhoA signalling through modulation of the function of Cav1.

PASMC proliferation is a prominent feature of PAH^1^,^4^. Hypoxia-induced PH has been shown to increase the activity of Rho/ROCK signalling and be attenuated by ROCK inhibitors^18^. We demonstrated that ROCK activity evaluated by MYPT1 phosphorylation in the lung of hypoxia-exposed *Murc*^−/−^ mice was weaker than that in the lung of hypoxia-exposed WT mice. cKO mice showed the alleviation of the development of hypoxia-induced PH and vascular remodelling. In addition, RhoA activity, proliferation and migration in MURC knockdown PASMCs and in *Murc*^−/−^ VSMCs were less than those in control cells. These findings suggest that the attenuation of PH in *Murc*^−/−^ and cKO mice is attributable to suppressed RhoA signalling in PASMCs. Previous studies demonstrated that *Cav1*^−/−^ mice developed PH^12^,^13^, and that proliferation, migration and RhoA activation were increased in *Cav1*^−/−^ VSMCs^11^,^51^. Taken together with MURC reversing the effects of Cav1 on RhoA activation in PASMCs, these observations suggest that Cav1 in PASMCs has a protective role in the development of PH, which contributes to the alleviation of PH in *Murc*^−/−^ and cKO mice.

The scaffolding domain of Cav1 is capable of switching off the activity of Gα-subunits^52^. We showed that both Cav1 and p115RhoGEF bind more efficiently to active Gα13 than to inactive Gα13, and that Cav1 inhibits the association of active Gα13 with p115RhoGEF, implying that the interaction of Cav1 with active Gα13 prevents further binding of active Gα13 to p115RhoGEF, which may account for the inhibitory effects of Cav1 on RhoA activity through the effects of Gα13. We also showed that the oligomerization and scaffolding domains of Cav1 bind to both Gα13 and MURC, suggesting that the competitive binding of MURC to Cav1 facilitates the association of Gα13 with p115RhoGEF, which leads to the activation of Rho/ROCK signalling. Various cellular functions ascribed to the small GTPase Rho are dependent on the spatial control of activation^45^. Inactive Rho is located within the cytoplasm and, following the activation of G-protein-coupled receptors, translocates to the plasma membrane, in which Rho is activated^43^,^49^. The subcellular localization of GEFs is a key aspect of Rho activity, and GEF activation is intimately linked with its localization^45^,^49^. Because MURC is associated with both p115RhoGEF and RhoA, our findings suggest that MURC functions as a scaffold to facilitate the compartmentation of p115RhoGEF and RhoA within the caveolae of PASMCs, which is likely to be involved in their activation and the subsequent activation of downstream signalling, which regulates the proliferation and migration of PASMCs. Collectively, our results suggest the novel concept that MURC functions as a component of caveolae to regulate Cav1-mediated Rho/ROCK signalling (Fig. 7e).

In addition to the Rho/ROCK pathway, Gα13 has been shown to be involved in several pathways, such as cadherin, PYK2 and apoptosis signal-regulating kinase-1 (ASK-1) signalling pathways^53^. Since MURC inhibits the association of Cav1 with Gα13, MURC may also influence signalling pathways mediated by Gα13. Cav1 deficiency and mutations cause PAH^6^,^12^,^13^. Therefore, alterations in the expression and function of Cav1 in PASMCs may generally contribute to the pathology of PAH. MURC modulates the effects of Cav1 in PASMCs; therefore, the inhibition of MURC may serve as a therapeutic target in PAH.

In conclusion, MURC deficiency in SMCs alleviates the development of PH. The association of MURC with Cav1 promotes the Gα13-mediated activation of p115RhoGEF, which leads to the subsequent activation of Rho/ROCK signalling, resulting in the enhanced proliferation and migration of PASMCs. Our findings provide a previously undescribed function for MURC in caveolar-mediated Rho/ROCK signalling and novel insights into the underlying pathogenetic mechanisms of PH.

## Methods

### Reagents

Rabbit polyclonal anti-Cav1 (1:500), anti-Gα13 (1:500) and anti-p115RhoGEF (1:50) antibodies were purchased from Santa Cruz Biotechnology; the rat monoclonal antibody to hemagglutinin (HA, 1:500) was from Roche Applied Science; the mouse monoclonal antibody to T7 (1:5,000) was from Novus Biologicals; the horseradish peroxidase-conjugated monoclonal antibody to GST (1:5,000) was from Wako Pure Chemical Industries, Ltd. (Osaka, Japan); the mouse monoclonal antibody to GAPDH (1:2,000) was from Millipore; the mouse monoclonal antibody to FLAG (1:1,000), Cy3-conjugated monoclonal antibody to αSMA (1:200), GDP and collagenase type 1 from *Clostridium histolyticum* were from Sigma-Aldrich; GTPγS was from Cytoskeleton, Inc.; the mouse monoclonal antibodies to Cav1 (1:200) and Cav3 (1:200) were from BD Biosciences; the rabbit polyclonal antibodies to MYPT1 (1:500), phospho-MYPT1 at Thr853 (pMYPT1^Thr853^, 1:500), and phospho-MLC2 at Ser19 (pMLC2^Ser19^, 1:500 for Western blotting, 1:50 for immunostaining) were from Cell Signaling Technology, Inc.; and the rabbit polyclonal antibody to Ki67 (1:100) and FITC-conjugated monoclonal antibody to αSMA (1:50) were from Abcam Biochemicals. The human Cav1- and Cav3-expressing vectors, pcDNA3.1-T7-hCav1 and pcDNA3.1-T7-hCav3, were gifts from Yukiko K. Hayashi (Tokyo Medical University, Tokyo, Japan)^27^. pcDNA3.1/nV5-p115RhoGEF(2A) and pCS2FLAG-human and p115RhoGEF (hp115RhoGEF) were provided by Keith Burridge (University of North Carolina) and Feng Shao (National Institute of Biological Sciences, Beijing, China), respectively. The human Gα13-expressing vector, pcDNA3.1-hGα13, and human Gα13(Q226L)-expressing vector, pcDNA3.1-hGα13(Q226L), were purchased from the Missouri S&T cDNA Resource Center. GST-Cav1(FL), GST-Cav1(1–61), GST-Cav1(61–101), and GST-Cav1(135–178) were gifts from William C. Sessa (Addgene plasmid #22445, 22446, 22447, and 22448). hPASMCs were purchased from PromoCell GmbH (Germany).

### Isolation and culture of SMCs

Rat VSMCs isolated from the thoracic aorta of Sprague-Dawley rats by the collagenase digestion method were maintained in DMEM with 10% FBS. In brief, the rat thoracic aorta was isolated under the thoracotomy. After the resection of connective tissue and fat tissue, the aorta was incubated with Hank's balanced salt solutions (HBSS) containing collagenase at 37 °C for 25 min. After the adventitia and intima were removed, the aorta was cut into 1 mm in the HBSS containing collagenase and elastase, and incubated at 37 °C for 3 h. The sample was suspended and centrifuged. The pellet was resuspended in 10 ml of fresh DMEM containing 10% FBS and incubated at 37 °C. Mouse VSMCs were isolated from the mouse aorta. In brief, the mouse aorta was isolated from the iliac bifurcation to the aortic arch by longitudinal incision. The aorta was incubated with DMEM containing 745 units per ml collagenase type 1 at 37 °C for 20 min. After washing the aorta with DMEM, the tissue was incubated with DMEM containing 280 units per ml collagenase and 11.7 units per ml elastase at 37 °C for 1 h. After filtration through a 40-μm filter, the filtrated sample was transferred to a 50-ml conical tube and centrifuged. The pellet was resuspended in 4 ml of fresh DMEM containing 10% FBS and incubated at 37 °C. Only early-passage (passage 3 or 4) cells were used in the experiments described below. hPASMCs were cultured in SmGM-2 medium (Lonza, Walkersville, MD, USA) supplemented with SmGM-2 SingleQuots (Lonza, Walkersville, MD, USA) according to the manufacturer's instructions.

### Production of a polyclonal antibody to MURC

Rabbit immunization was conducted by UNITECH (Chiba, Japan) using synthetic peptides corresponding to the N-terminal residues of mouse Murc (MEHNGSASNAGKIHQNRC). In the Western blot analysis, IgG was purified from antisera.

### Immunostaining

Specimens from the lung were fixed in 4% PFA/phosphate-buffered saline (PBS) and stained with the Cy3-conjugated αSMA antibody (1:200), FITC-conjugated αSMA antibody (1:50), pMLC2^Ser19^ antibody (1:50), or Ki67 antibody (1:50). Secondary antibodies were conjugated with Alexa Fluor 488 or 555 (1:500), and nuclei were visualized using DAPI (1:1,000). hPASMCs were fixed with 4% PFA/PBS at room temperature for 10 min and stained with the MURC (1:200), Cav1 (1:200), Cav3 (1:200) or p115RhoGEF (1:50) antibody. Fluorescent signals were detected using a Zeiss LSM510 META Confocal Imaging System (Carl Zeiss).

### Plasmid constructs

Complemetnary DNA (cDNA) encoding human MURC/Cavin-4 with a C-terminal anti-DYKDDDDK (FLAG) or HA epitope were cloned into pcDNA3.1 (Invitrogen) to generate pcDNA3.1-MURC-FLAG or -HA, respectively^24^,^34^. The cDNA fragment corresponding to human RhoA (hRhoA) with a C-terminal HA epitope was cloned by a polymerase chain reaction (PCR) from a human heart cDNA library (Stratagene) using a forward primer (5′-CACCATGGCTGCCATCCGGAAGAA-3′) and reverse primer (5′-AGCGTAATCTGGAACATCGTATGGGTACAAGACAAGGCACCCAGATTTTTTCTTCCC-3′), and hRhoA-HA fragments were then inserted into pcDNA3.1D/V5-His-TOPO (Invitrogen) to generate pcDNA3.1-hRhoA-HA. The cDNA fragment of human Gα13 was digested from pcDNA3.1-hGα13 and inserted into pGEX-6P-2 (GE Healthcare) to generate pGEX-Gα13. The corresponding cDNA fragments for mouse MURC (mMURC) were cloned by PCR with a mouse heart cDNA template. PCR was performed using the following primers: MURC, forward primer 5′-ATG GAA CAC AAC GGA TCA GCT-3′ and reverse primer 5′-CTA TTT GTA GTC TGA GGA CTG CTT TAG CTC CA-3′. cDNA encoding MURC with a COOH-terminal Flag epitope and LacZ were cloned into the pMSCVpuro Retroviral Vector (Clontech) to generate pMSCVpuro-MURC and pMSCVpuro-LacZ, respectively.^20^,^21^. cDNA encoding hCav1 with a C-terminal HA epitope was cloned by PCR with pcDNA3.1-T7-hCav1 using a forward primer (5′-GGAGATCTATGTCTGGGGGCAAATACGT-3′) and reverse primer (5′-GGGGTTAACTTAAGCGTAATCTGGAACATCGTATGGGTATATTTCTTTCTGCAAGTTGATGCGG-3′). The hCav1-HA and LacZ fragments were inserted into the pMSCVhyg Retroviral Vector to generate pMSCVhyg-hCav1-HA and pMSCVhyg-LacZ, respectively. The human p115RhoGEF(2A) fragment digested from pcDNA3.1/nV5-p115RhoGEF(2A) was inserted into the pMSCVhyg Retroviral Vector (Clontech) to generate pMSCVhyg-p115RhoGEF(2A). The cDNA fragments corresponding to hMURC, hCav1, hCav3, and hp115RhoGEF without a stop codon were cloned by PCR with pcDNA3-hMURC, pcDNA3.1-T7-hCav1, pcDNA3.1-T7-hCav3, and pCS2FLAG-hp115RhoGEF as templates with the following primers, respectively: hMURC forward primer (5′-AACGGATCCATGGAACATAATGGGTCTGC-3′) and hMURC reverse primer (5′-GGCCGAATTCTTGTCGTCATCGTCTTTGT-3′); hCav1 forward primer (5′-CTATGGATCCATGTCTGGGGGCAAATACGT-3′) and hCav1 reverse primer (5′-GGGAATTCTCTATTTCTTTCTGCAAGTTGA-3′); hCav3 forward primer (5'-CTATGGATCCATGATGCCAGAAGAGCACAC-3') and hCav3 reverse primer (5′-GTTTGAATTCTCAGCGTAATCTGGAACATCGTATGGGTAGACCTCCTTCCGCAGCACCA-3′); and hp115RhoGEF forward primer (5′-AAAAGCTTATGGAAGACTTCGCCCGAGGGGCGGC-3′) and hp115RhoGEF reverse primer (5′-AGCTCCTCCAAGGGGAAGAACAGGCATTCT-3′). To construct mammalian expression vectors for BiFC assays, the cDNA fragments of hMURC, hCav1, hCav3 and hp115RhoGEF were fused to N- or C-terminal portions of the divided monomeric Kusabira-Green (mKG) fragments of phmKGN-MC, phmKGC-MC, phmKGN-MN and phmKGC-MN (Medical & Biological Laboratories, Nagoya, Japan) to generate phmKGN-MC-hCav1, phmKGC-MC-hMURC, phmKGC-MN-hCav3, phmKGN-MN-hMURC, phmKGN-MC-hMURC, and phmKGC-MN-hp115RhoGEF.

### Immunoprecipitation

COS cells transfected with each of the plasmids using FuGENE6 (Roche Applied Science) were washed with ice-cold PBS and lysed with lysis buffer (50 mM Tris-HCl, pH 8.0, 50 mM NaCl, 1% Nonidet P-40) containing protease inhibitory cocktail (Pierce), 1 mM Na_3_VO_4_, 1 mM NaF, 1 mM phenylmethylsulfonyl fluoride, and 60 mM octyl glucoside. Immunoprecipitation was carried out by incubating the same amount of cell lysates with magnetic beads (Magnosphere MS300/Carboxyl, COSMO BIO, Tokyo, Japan) coated with each antibody at 4 °C overnight. Beads were washed with wash buffer (50 mM Tris, pH 8.0, 50 mM NaCl, 1.0% Nonidet P-40, 1 mM NaF) five times and the precipitated proteins were separated by SDS–PAGE, transferred to a polyvinylidene difluoride membrane, and probed with each antibody.

### Western blotting

Total cell lysates or fractionated lysates were electrophoresed in SDS–PAGE and transferred to polyvinylidene difluoride membranes. The peroxidase-conjugated anti-GST antibody was used for the immunoblotting of GST. To separate the membrane fraction and cytosol fraction, mouse VSMCs were harvested with PBS and resuspended with phosphate buffer, pH 6.8 (0.1 M NaH_2_PO_4_, 0.1 M Na_2_HPO_4_, 8.5% sucrose, protease inhibitor cocktail, 1 mM Na_3_VO_4_, and 1 mM NaF). Cells were homogenized and centrifuged at 1,000 *g* at 4 °C for 10 min. Supernatants were ultracentrifuged at 55,000 r.p.m. at 4 °C for 30 min. Pellets were lysed and loaded as the membrane fraction and supernatant as the cytosol fraction.

### Protein–protein interaction analysis

In the *in situ* protein association analysis, we performed BiFC assays using a CoralHue Fluo-chase Kit (Medical & Biological Laboratories) according to the manufacturer's protocol^34^,^54^,^55^. In each protein association assay, the appropriate pairs of plasmids in which each target protein gene was fused to the divided CoralHue Kusabira-Green gene N-terminal fragment or C-terminal fragment were cotransfected into the cultured hPASMCs. After a 24-h incubation, the fluorescent signal was detected using a Zeiss LSM510 META Confocal Imaging System.

### RT-quantitative PCR

Total RNA was extracted from cells or tissues using an RNeasy Mini kit (Qiagen) or TRIzol reagent (Invitrogen) and then treated with DNase I (Qiagen) to remove any residual DNA. Total RNA was converted to cDNA using the High Capacity cDNA Reverse Transcription Kit (Applied Biosystems). Synthesized cDNA was analysed by kinetic real-time PCR using Takara PCR Thermal Cycler Dice (Takara Bio) with Platinum SYBR Green qPCR Super Mix (Invitrogen)^20^,^34^,^56^. The primer sequences used were as follows: mouse *Murc* forward primer (5′-ACAGTCACACAGCAATACGGGCTA-3′) and mouse *Murc* reverse primer (5′-TTCTCGGGCAGGCTTCTGTCTTTA-3′); and mouse *GAPDH* forward primer (5′-TTGTGATGGGTGTGAACCACGAGA-3′) and mouse *GAPDH* reverse primer (5′-CATGAGCCCTTCCACAATGCCAAA-3′).

### Animals

*Murc*^−/−^ and *Murc* floxed (*Murc*^*fl/+*^) mice (C57BL/6J background) have been obtained using Cre-loxP and FLP-FRT system^34^. To generate *Murc* conditional knockout (*SM22Cre*^*+*^*Murc*^*fl/fl*^) mice, *Murc*^*fl/fl*^ mice were bred with *SM22Cre* transgenic mice expressing Cre recombinase under the transcriptional control of the SMC-restricted *SM22* promoter (*SM22Cre*) (C57BL/6J background, Jackson Laboratory). Inheritance of the *SM22Cre* transgene was determined by PCR, using the following primers: forward (5′-TACTCTCCTTCCAGTCCACAAAC-3′) and reverse (5′-AGGTAGTTATTCGGATCATCAGCTA-3′). *Cav1*-null (*Cav1*^−/−^) mice (C57BL/6J background) were purchased from Jackson Laboratory. Male mice aged 12 weeks were used in this study. All of the aspects of animal care and experimentation performed in this study were approved by the Institutional Animal Care and Use Committee of Kyoto Prefectural University of Medicine.

### Hypoxic mouse model of PH

WT and *Murc*^−/−^ mice, or *Murc*^*fl/fl*^ and cKO mice were exposed to normobaric hypoxia (10% O_2_) in a chamber in which oxygen was tightly regulated by the oxygen controller ProOx110 (KYODO International, Kanagawa, Japan) for 4 weeks^57^. Nitrogen was automatically introduced as required to maintain the proper fraction of inspired oxygen (FiO_2_). Age- and sex-matched littermates were exposed to identical conditions in normoxia and served as controls.

### Measurement of RV pressure and histological analyses

Mice were intubated with a 22-gauge Teflon tube and placed in a supine position. To measure RV hemodynamics, open-chest RV catheterization using a 1.2-F pressure catheter (Scisense Inc.) was performed during anaesthesia with 1.5% isoflurane. Pulmonary vascular remodelling was assessed by measuring the medial thickness of alveolar/distal pulmonary vessels of 25–100 μm in diameter, which are not associated with bronchi, from lung sections immunostained with αSMA. Per cent wall thickness is expressed as the medial wall area (the area between the internal and external lamina) divided by the area of the vessel (the area between the external lamina). Multiple lung sections were made for each mouse and >5 vessels were analysed in each lung section.

### Insertion of an osmotic pump

Mice were anaesthetized with 1.5% isoflurane, the skin was shaved, and mini-osmotic pumps (model 2004; Alzet, Cupertino, CA, USA) containing either PBS or Y-27632 (CALBIOCHEM; 125 mg ml^−1^ of saline) were inserted into a subcutaneous pocket through a small incision made in the skin between the scapulae. These pumps delivered a volume of 0.25 μl h^−1^, which was equivalent to 30 mg kg^−1^·day^−1^ of Y-27632 for a 25-g mouse^58^.

### Histological analysis

Fixation was performed using 4% PFA/PBS. Hearts, lungs and aortae were cut and paraffin sections of 3 μm thick were stained with H&E.

### Transmission electron microscopy and quantitation

Twelve-week-old mouse lungs were fixed with 2% glutaraldehyde in 0.1 M cacodylate buffer, post-fixed with 2% OsO_4_, and stained with uranyl acetate and lead citrate. Microtome sections were examined under a H-7100 transmission electron microscope (HITACHI, Tokyo, Japan) and photographed at a magnification of × 40,000 or × 20,000. Caveolae were identified by their characteristic flask shape and location at or near the plasma membrane^34^,^56^.

### Echocardiographic and morphometric analyses

After mice had been anaesthetized with isoflurane (1.5%, Abbott Laboratories), echocardiography was performed using a Vevo 2100 system (VisualSonics) equipped with a 30-MHz microprobe. After echocardiography, mice were sacrificed by cervical dislocation and then weighed. Hearts were excised, rinsed in PBS and weighed. Body weight and left tibial length were measured to normalize heart weight.

### Gene silencing through RNA

Human MURC-specific and control siRNA duplex oligonucleotides (Stealth RNA interface (RNAi)) were purchased from Invitrogen. siRNAs were transiently transfected into hPASMCs using Lipofectamine RNAiMAX reagent (Invitrogen) according to the manufacturer's protocol. The medium was changed 4 h post-transfection and cells were used for assay 72 h after transfection. The siRNA sequences were as follows: *MURC* siRNA-1 (sense (S), 5′-CCGUCCAGAUUGACCUGUUGAAGCU-3′; antisense (AS), 5′-AGCUUCAACAGGUCAAUCUGGACGG-3′); *MURC* siRNA-2 (S, 5′-GGAAGUCAGGCAAGGAGCACAUUGA-3′; AS, 5′-UCAAUGUGCUCCUUGCCUGACUUCC-3′); *p115RhoGEF* siRNA-1 (sense (S), 5'-GCCGUGAGAUUCUGCACCACGUUAA-3'; antisense (AS), 5′-UUAACGUGGUGCAGAAUCUCACGGC-3′); and *p115RhoGEF* siRNA-2 (S, 5′-CCCUGUUCCUCGAUCGCUUGAUGAA-3′; AS, 5′-UUCAUCAAGCGAUCGAGGAACAGGG-3′).

### Recombinant retroviruses and gene transfer

To generate recombinant retroviruses, GP2–293 cells (Clontech) were cotransfected with the helper vector pVSV-G and pMSCVpuro-LacZ, pMSCVhyg-LacZ, pMSCVpuro-Murc-FLAG or pMSCVhyg-hCav1-HA. The medium supernatant was collected and centrifuged to concentrate virus stocks according to the manufacturer's instructions. hPASMCs were infected with the retrovirus in the presence of 8 μg ml^−1^ polybrene for 24 h, and medium was changed to fresh medium. Infected cells were selected with 1 μg ml^−1^ puromycin or 50 μg ml^−1^ hygromycin.

### Proliferation assay

Cell proliferation was assessed using cell proliferation reagent WST-1 (4-[3-(4-iodophenyl)-2-(4-nitrophenyl)-2H-5-tetrazolio]-1,3-benzene disulfonate) (Roche Applied Science). In brief, 5,000 cells were seeded on 96-well plates and incubated in DMEM containing 0.4% FBS for mouse VSMCs at 37 °C for 48 h, or in growth medium containing 5% FBS with or without Y-27632 for hPASMCs at 37 °C for 24 h. WST-1 reagent was added and the cells were incubated at 37 °C for 4 h. The absorbance of the samples was measured using a microplate reader at 450 nm.

### Migration assay

Confluent monolayer cells in a 60-mm dish were scraped in a straight line to create a ‘scratch' with a p200 pipette tip. The scratched cells were washed once with DMEM to remove debris and smooth the edge of the scratch, and then replaced with 4 ml of migration medium (DMEM with 0.1% BSA). The dish was incubated at 37 °C for 24 h. Wound closure was quantified by the per cent change in the wound area. A Boyden chamber assay was performed using a Transwell Permeable Support 8.0-μm polycarbonate membrane (Coster). The lower chamber contained 1% FBS as a chemoattractant. hPASMCs transfected with control siRNA or *MURC* siRNAs were prepared in serum-free medium, and 3 × 10^4^ cells were added to the upper chamber. After a 4-h incubation, migrated cells were stained with Hoechst 33342 and counted under a microscope^59^,^60^,^61^,^62^.

### GST pulldown assay

Ten micrograms of GST-Cav1(FL), GST-Cav1(1–61), GST-Cav1(61–101), or GST-Cav1(135–178) was bound to glutathione-Sepharose beads. The beads were incubated with lysates from COS cells transfected with the MURC or Gα13 expression plasmid at 4 °C for 2 h. GST-conjugated glutathione-Sepharose beads were used as a control. Beads were washed five times, and proteins were eluted for western blotting. In the GST pulldown assay of the Gα13 protein, 10 μg of the GST-Gα13 protein was preloaded with GDP (1 mM) or GTPγS (0.1 mM) to binding buffer (0.1 M Tris-HCl, pH 7.4, 1 mM EDTA, 2 mM DTT, 0.2 M NaCl) and incubated at 30 °C for 15 min. After adding a stop buffer (50 mM MgCl_2_), the GST-Gα13 protein converted to the inactive or active form was linked to glutathione-Sepharose beads, which were then incubated with lysates from COS cells transfected with T7-Cav1, MURC-HA, and/or p115RhoGEF-FLAG expression plasmids at 4 °C for 2 h.

### Statistical analyses

All experiments were performed at least three times. Data are expressed as means±standard errors. Data were analysed by the unpaired Student's *t*-test for comparisons between two groups or a one-way analysis of variance with a *post hoc* analysis for multiple comparisons. A *P* value of <0.05 was considered significant.

### Data availability

The authors declare that all the data supporting the findings of this study are available within the article and its Supplementary Information Files and from the authors upon request.

## Additional information

**How to cite this article:** Nakanishi, N. *et al.* MURC deficiency in smooth muscle attenuates pulmonary hypertension. *Nat. Commun.* 7:12417 doi: 10.1038/ncomms12417 (2016).

## Supplementary Material

Supplementary Information Supplementary Figures 1-6 and Supplementary Tables 1-3
