# Supplementary material for: MURC deficiency in smooth muscle attenuates pulmonary hypertension
Source: Nat Commun. 2016 Aug 22;7:12417. doi: 10.1038/ncomms12417 (PMC4996946; doi:10.1038/ncomms12417)
Supplement: Supplementary Information — Supplementary Figures 1-6 and Supplementary Tables 1-3 [file ncomms12417-s1.pdf]

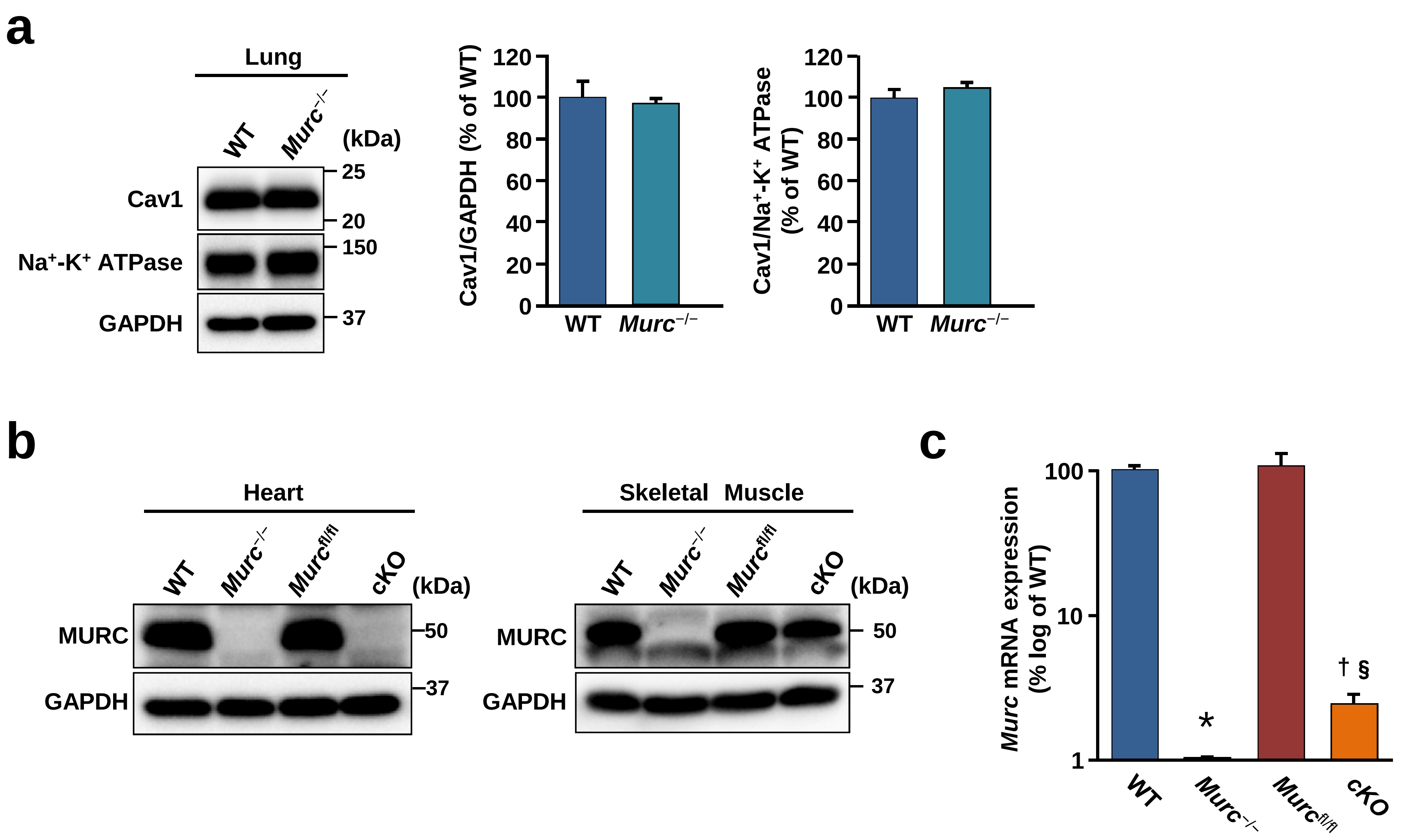

a
Lung
WT
Murc−/−
(kDa)
Cav1
Na+-K+ ATPase
GAPDH
25
20
150
37
Cav1/GAPDH (% of WT)
0
20
40
60
80
100
120
WT
Murc−/−
Cav1/Na+-K+ ATPase (% of WT)
b
Heart
Skeletal Muscle
Murcfl/fl
cKO
MURC
50
c
Murc mRNA expression (% log of WT)
1
10
*
† §

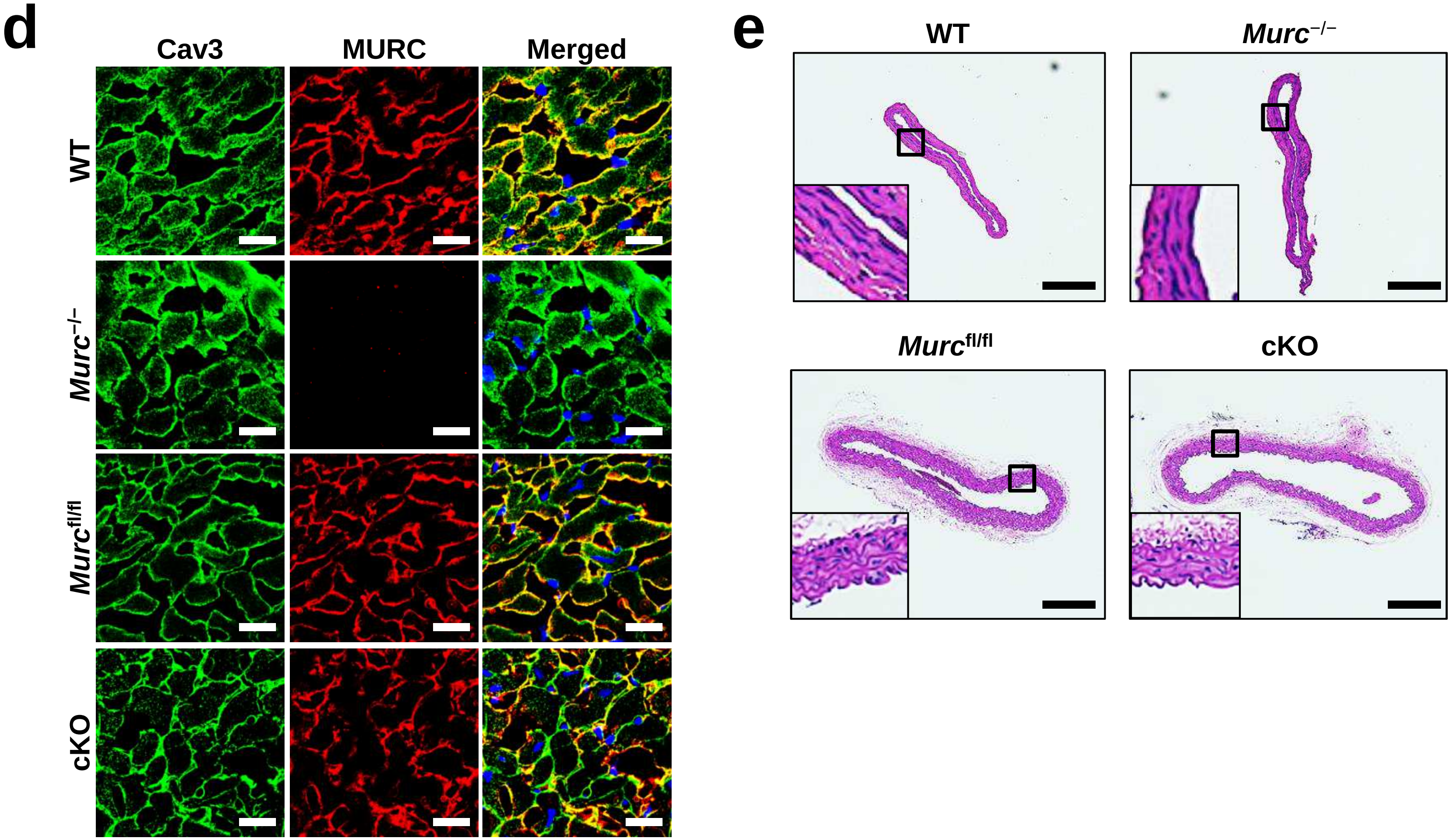

Supplementary Figure 1. The heart, skeletal muscle, and aorta in cKO mice.

(a) Cav1 protein expression in WT in *Murc*$^{-/-}$ lungs. Cav1 protein expression in *Murc*$^{-/-}$ lungs was not significantly different from that in WT lungs (n = 3 per group). (b) Murc protein expression was examined in the heart and skeletal muscle of WT, *Murc*$^{-/-}$, *Murc*$^{fl/fl}$, and cKO mice. (c) Murc mRNA expression was examined in the heart and skeletal muscle of WT, *Murc*$^{-/-}$, *Murc*$^{fl/fl}$, and cKO mice (n = 3 per group). *$P<0.05$ compared with WT mice, $^{\dagger}P<0.05$ compared with *Murc*$^{fl/fl}$ mice, $^{\S}P<0.05$ compared with *Murc*$^{-/-}$ mice. (d) Representative immunostaining images of Murc and Cav3 in the heart of WT, *Murc*$^{-/-}$, *Murc*$^{fl/fl}$, and cKO mice. Bar, 20 μm. (e) Representative H & E staining images of the aorta in WT, *Murc*$^{-/-}$, *Murc*$^{fl/fl}$, and cKO mice. Bar, 200 μm. Data are presented as mean ± SEM. Uncropped images of blots are shown in Supplementary Fig. 6.

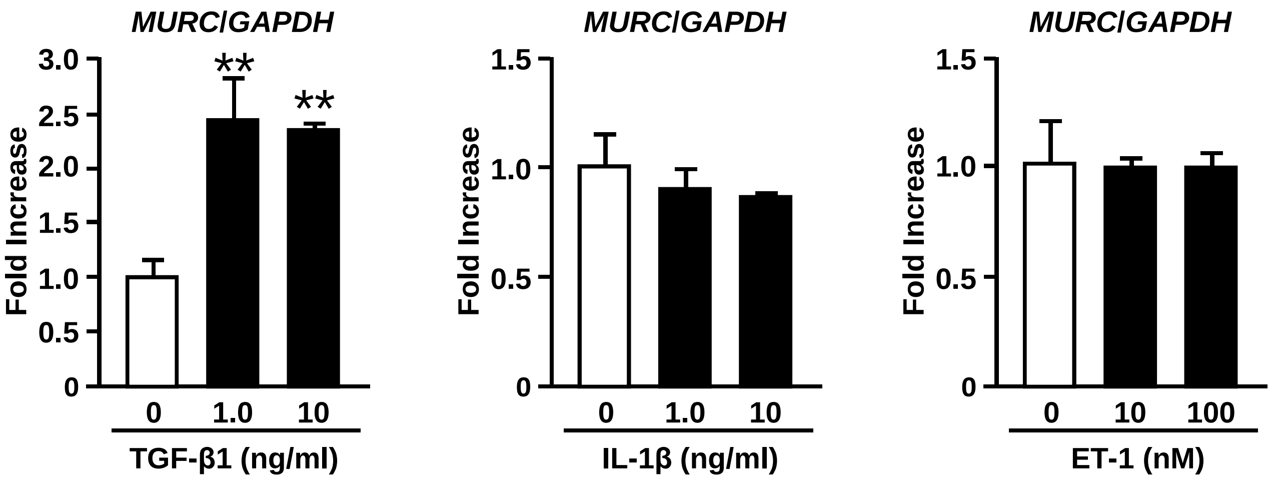

Supplementary Figure 2. Induction of *MURC* mRNA expression by TGF-β1 in hPASMCs.

hPASMCs were treated with TGF-β1 for 24 hrs, IL-1β for 24 hrs, or ET-1 for 16 hrs (n = 3 per group). ***P*<0.01 compared with hPASMCs without a stimulation. Data are presented as mean ± SEM.

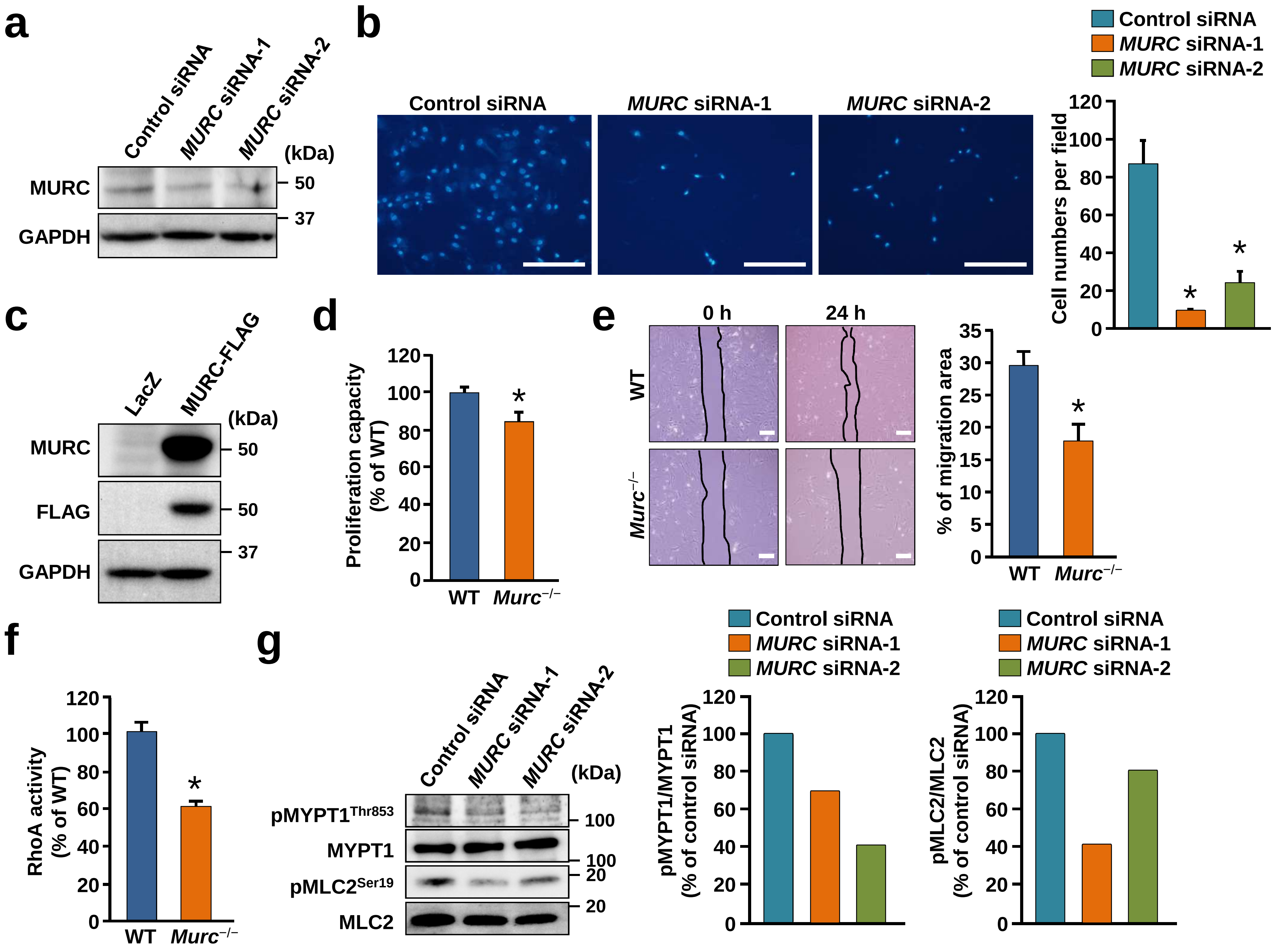
a
Control siRNA
MURC siRNA-1
MURC siRNA-2
(kDa)
MURC
GAPDH
50
37
b
Control siRNA
MURC siRNA-1
MURC siRNA-2
Cell numbers per field
c
LacZ
MURC-FLAG
(kDa)
MURC
FLAG
GAPDH
d
Proliferation capacity (% of WT)
WT
Murc−/−
e
0 h
24 h
WT
Murc−/−
% of migration area
f
RhoA activity (% of WT)
g
pMYPT1Thr853
MYPT1
pMLC2Ser19
MLC2
100
20
pMYPT1/MYPT1 (% of control siRNA)
pMLC2/MLC2 (% of control siRNA)

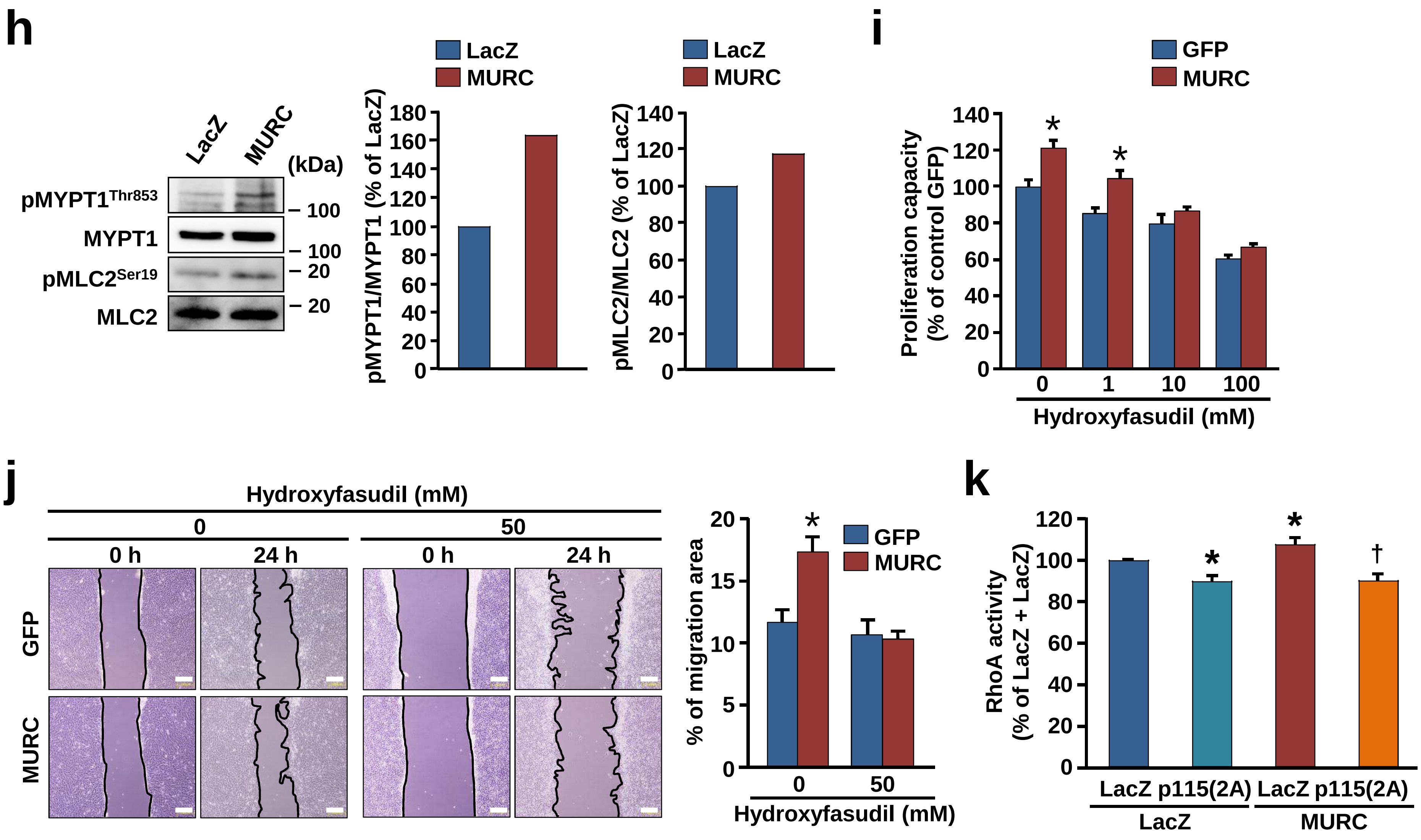
h
LacZ
MURC
(kDa)
pMYPT1Thr853
MYPT1
pMLC2Ser19
MLC2
100
100
20
20
pMYPT1/MYPT1 (% of LacZ)
180
160
140
120
100
80
60
40
20
0
pMLC2/MLC2 (% of LacZ)
140
120
100
80
60
40
20
0
i
GFP
MURC
Proliferation capacity
(% of control GFP)
140
120
100
80
60
40
20
0
*
*
0
1
10
100
Hydroxyfasudil (mM)
j
Hydroxyfasudil (mM)
0
50
0 h
24 h
0 h
24 h
GFP
MURC
% of migration area
20
15
10
5
0
*
0
50
Hydroxyfasudil (mM)
k
RhoA activity
(% of LacZ + LacZ)
120
100
80
60
40
20
0
*
*
†
LacZ p115(2A) LacZ p115(2A)
LacZ
MURC

Supplementary Figure 3. Attenuation of proliferation, migration, and RhoA activity in MURC-deficient SMCs.

(a) Lysates from hPASMCs transfected with control siRNA or *MURC* siRNA were immunoblotted with an anti-MURC antibody. (b) Left, representative images of a transwell migration assay using hPASMCs transfected with control siRNA or *MURC* siRNA. Bar, 200 μm. Right, quantification of migrated hPASMCs (n = 8 per group). *P<0.05 compared with control siRNA. (c) Lysates from hPASMCs transduced with a retrovirus expressing LacZ or MURC-FLAG were immunoblotted with anti-MURC and anti-FLAG antibodies. (d) The proliferation capacities of WT and $Murc^{-/-}$ VSMCs treated with FBS were assessed using a WST-1 cell proliferation assay system (n = 3 per ggroup). *P<0.05 compared with WT VSMCs. (e) The migration of WT and $Murc^{-/-}$ VSMCs was assessed by a wound healing assay (n = 4 per group). Wound closure was quantified by the percent change in the wound area. Bar, 100 μm. *P<0.05 compared with WT VSMCs. (f) RhoA activity was measured in WT and $Murc^{-/-}$ VSMCs (n = 3 per group). Starved cells were stimulated with 1% FBS for 1 h. *P<0.05 compared with WT VSMCs. (g) The phosphorylation of MYPT1 and MLC2 was assessed in hPASMCs transfected with control siRNA or *MURC* siRNAs. Starved cells were stimulated with 1% FBS for 1 h. (h) The phosphorylation of MYPT1 and MLC2 was assessed in LacZ- and MURC-overexpressing hPASMCs. (i) Attenuation of MURC-induced proliferation in VSMCs by the ROCK inhibitor, hydroxyfasudil. The proliferation capacities of GFP- and MURC-overexpressing rat VSMCs were assessed using a WST-1 cell proliferation assay system (n = 3-4 per group). *P<0.05 compared with GFP-overexpressing VSMCs. (j) Attenuation of MURC-induced migration in VSMCs by the ROCK inhibitor, hydroxyfasudil. The migration capacities of GFP- and MURC-overexpressing rat VSMCs were assessed by a wound healing assay (n = 3-4 per group). Wound closure was quantified by the percent change in the wound area. *P<0.05 compared with GFP-overexpressing VSMCs. (k) RhoA activity was assessed in hPASMCs transduced with LacZ + LacZ, LacZ + p115RhoGEF(2A) [p115(2A)], MURC-FLAG + LacZ, and MURC-FLAG + p115(2A) (n = 7 per group). hPASMCs were infected with a puromycin-resistant retrovirus expressing LacZ and MURC-FLAG. After being selected using puromycin, hPASMCs were infected with a hygromycin-resistant retrovirus expressing LacZ and p115(2A), and subsequently selected using hygromycin. *P<0.05 compared with LacZ + LacZ, $^{\dagger}P$<0.05 compared with MURC + LacZ. Data are presented as mean ± SEM. Uncropped images of blots are shown in Supplementary Fig. 6.

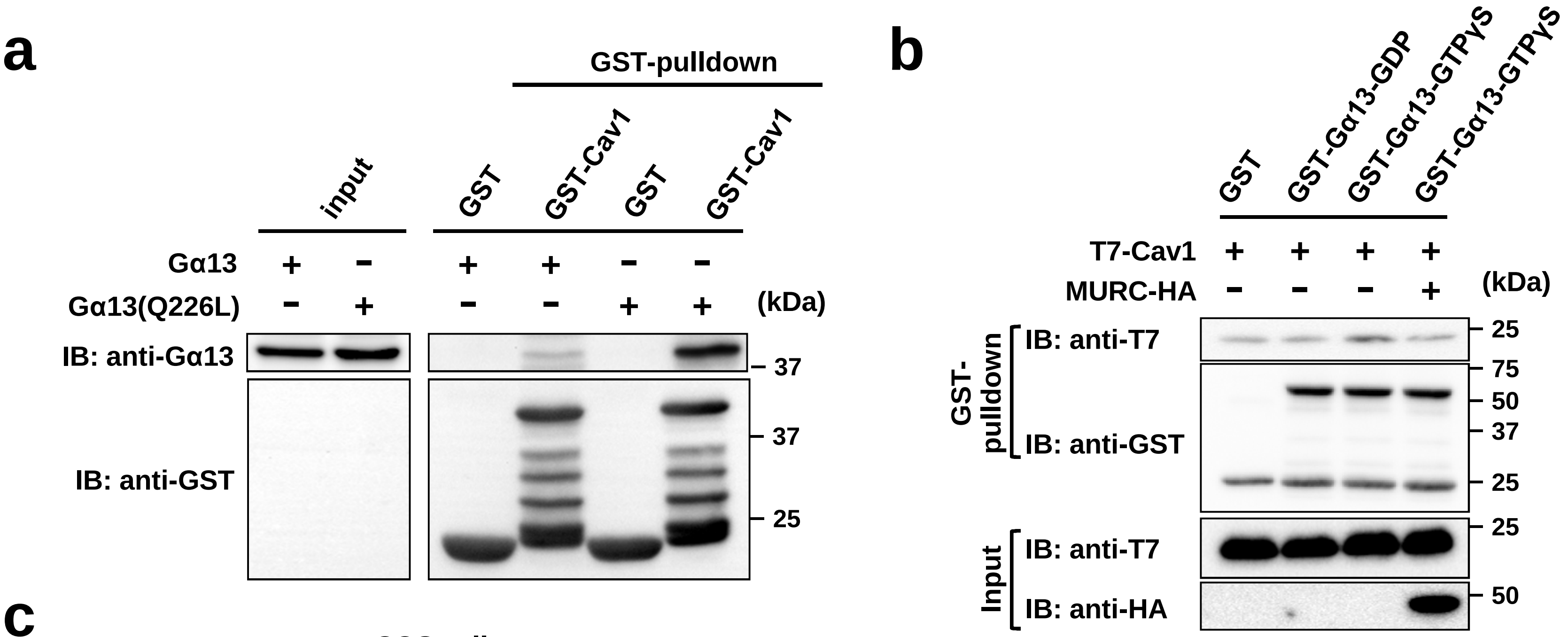

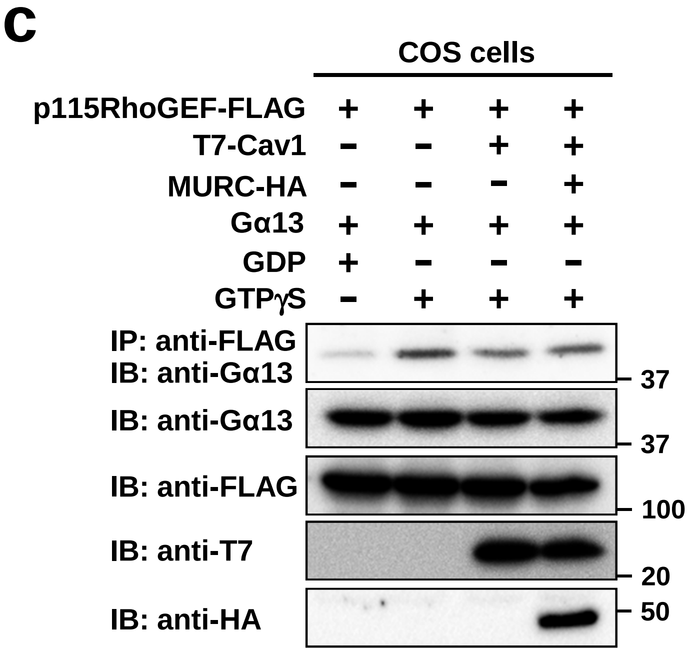

Supplementary Figure 4. Inhibition of the association of Cav1 with Gα13 by MURC.

(a) COS cells were transfected with plasmids expressing Gα13 and Gα13(Q226L). GST pulldown was performed with GST fusion Cav1 conjugated to glutathione-Sepharose beads and the COS cell lysates. Precipitated proteins were blotted with anti-Gα13 and anti-GST antibodies. (b) GST fusion Gα13 conjugated to glutathione-Sepharose beads was preloaded with GDP or GTPγS. GST pulldown was performed with COS cell lysates transfected with plasmids expressing the indicated proteins. Precipitated proteins were blotted with anti-T7 and anti-GST antibodies. (c) COS cells were transfected with pCS2FLAG-hp115RhoGEF and/or pcDNA3.1-T7-hCav1, pcDNA3.1-hMURC-HA, and pcDNA3.1-hGα13, and cell lysates were immunoprecipitated with the anti-FLAG antibody. Uncropped images of blots are shown in Supplementary Fig. 6.

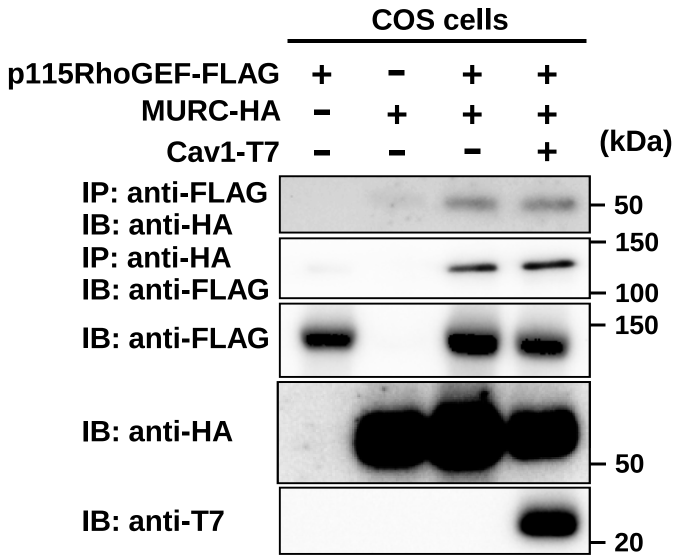

Supplementary Figure 5. Association of p115RhoGEF with MURC.

COS cells were transfected with pCS2FLAG-hp115RhoGEF, pcDNA3.1-hMURC-HA, and/or pcDNA3.1-T7-hCav1. Cell lysates were immunoprecipitated with anti-FLAG and anti-HA antibodies. Uncropped images of blots are shown in Supplementary Fig. 6.

Fig. 1b

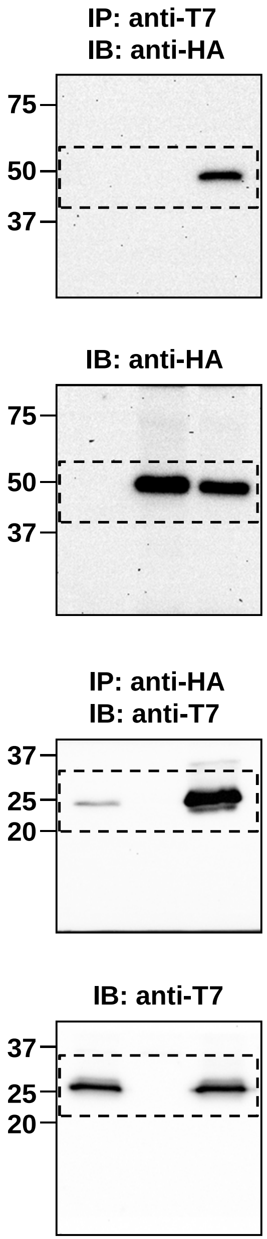

Fig. 1c

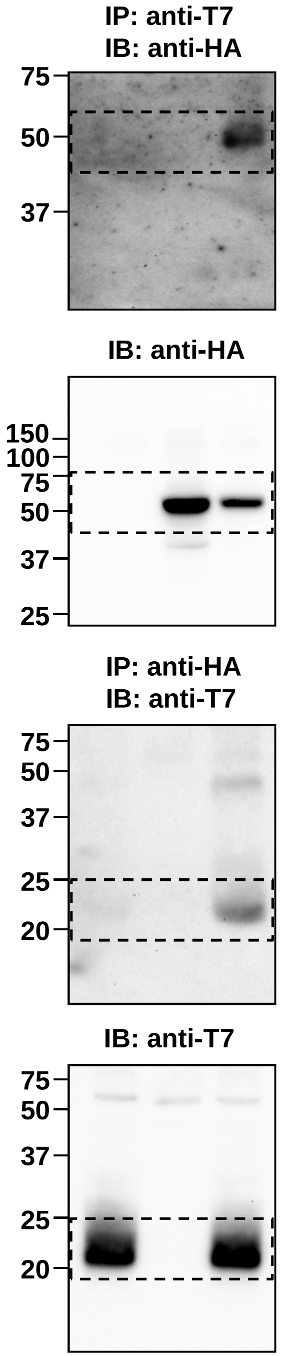

Fig. 2b

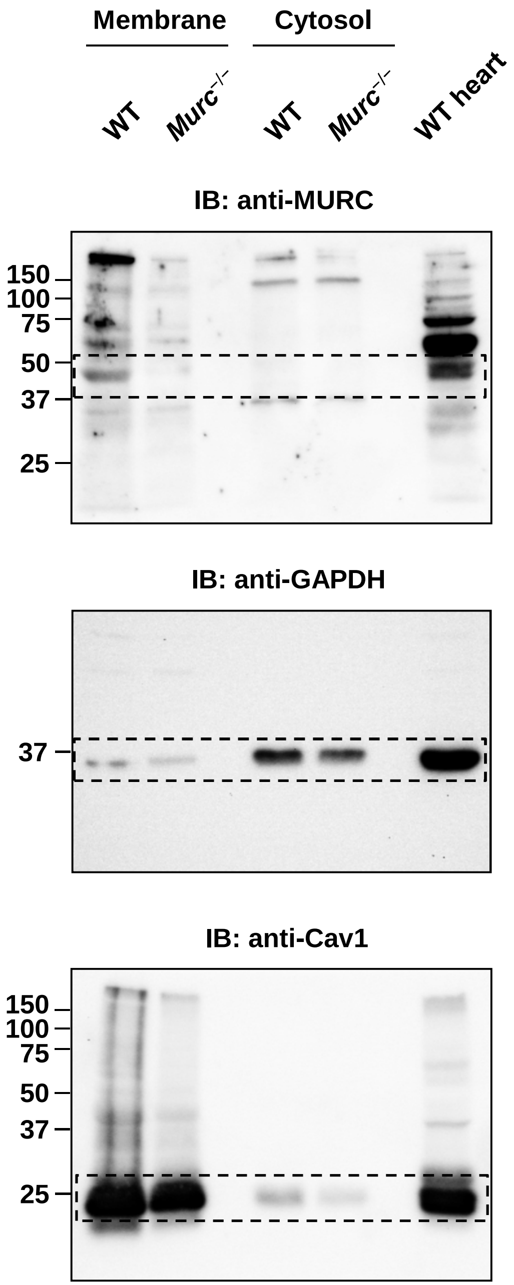

Fig. 3a

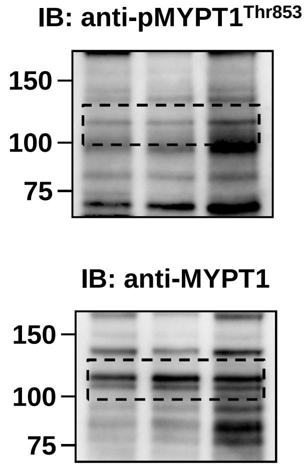

Fig. 3c

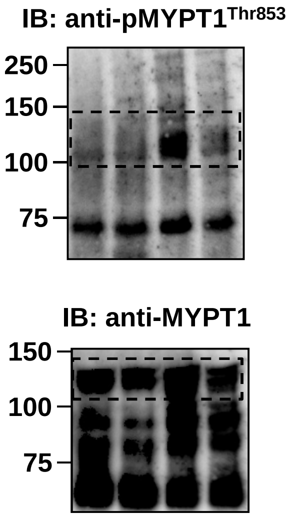

Fig. 3e

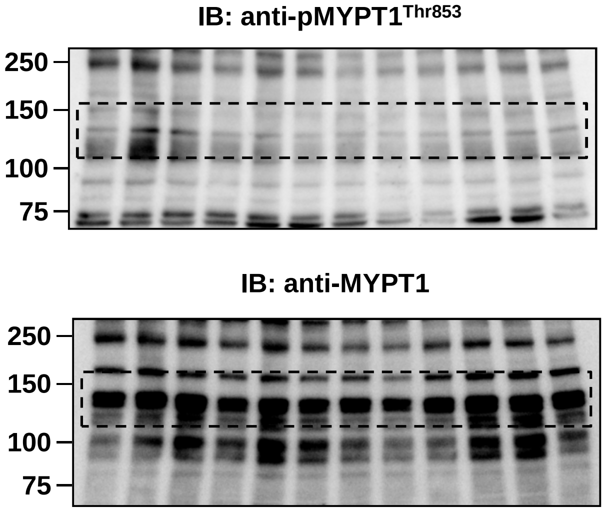

Fig. 3f

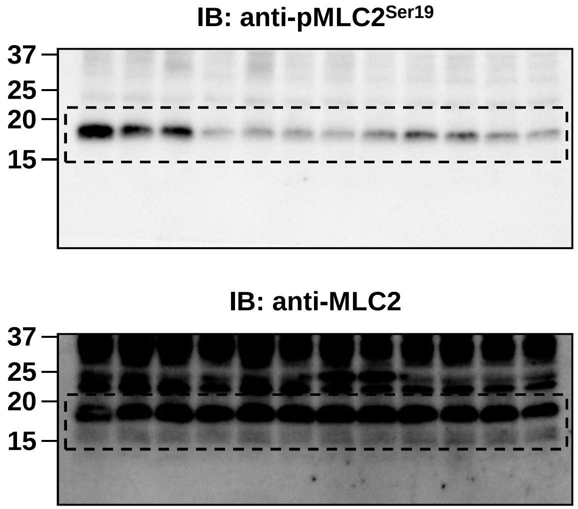

Fig. 4a

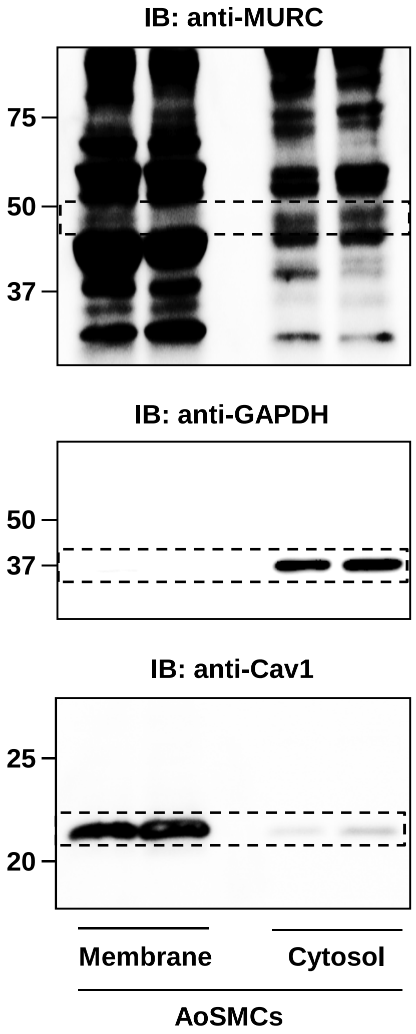

Fig. 6a

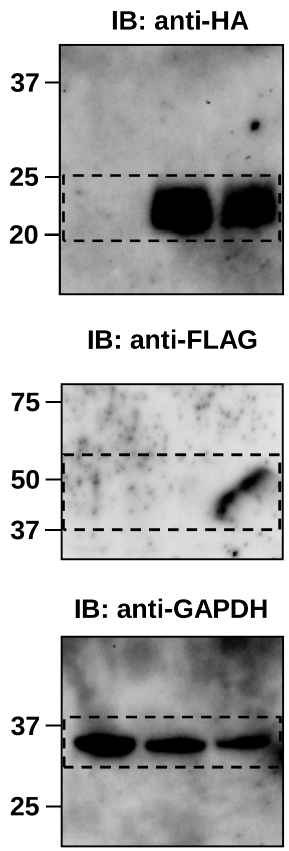

Fig. 6b

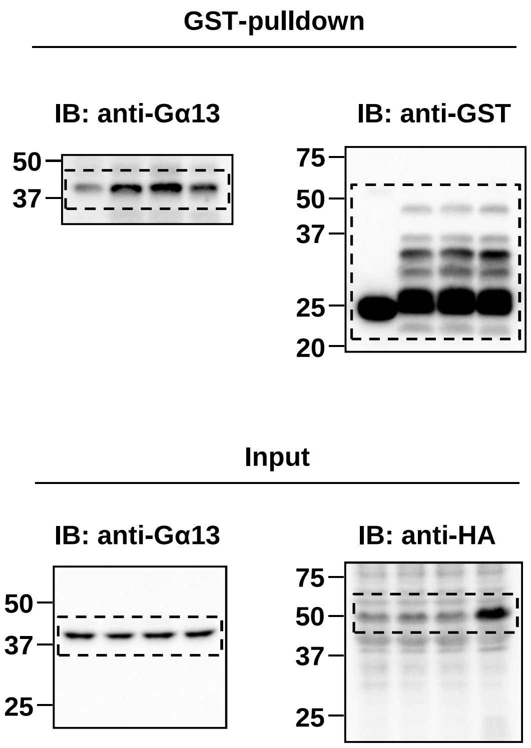

Fig. 6c

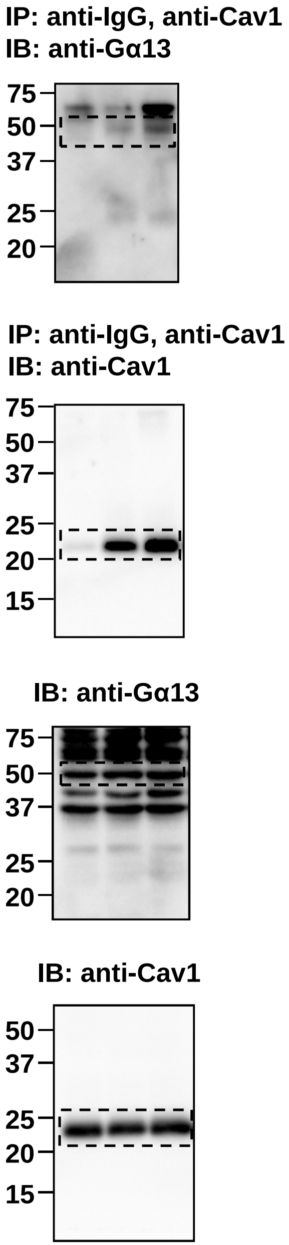

Fig. 6d

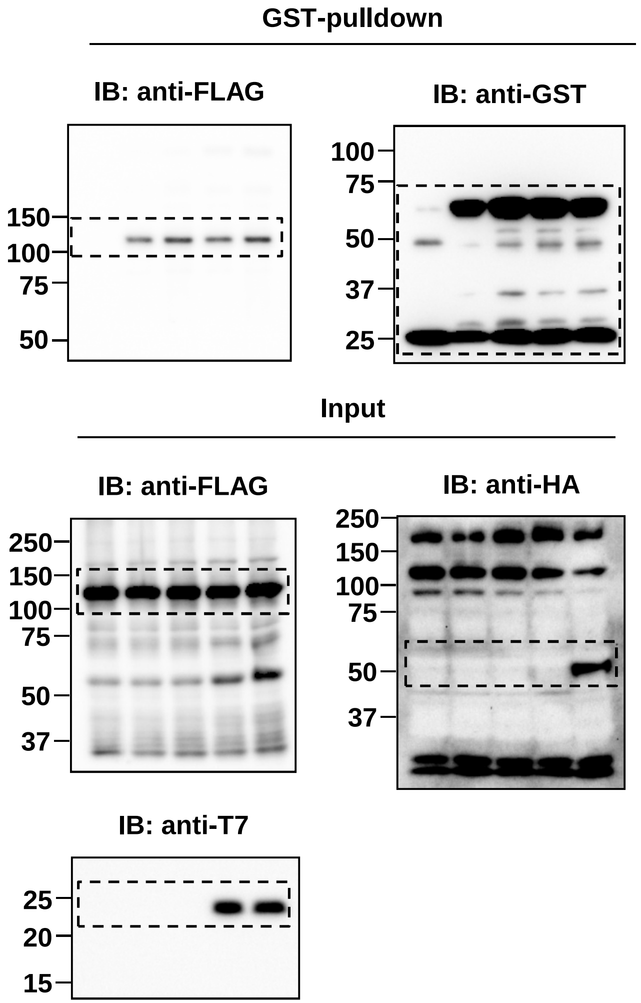

Fig. 6e

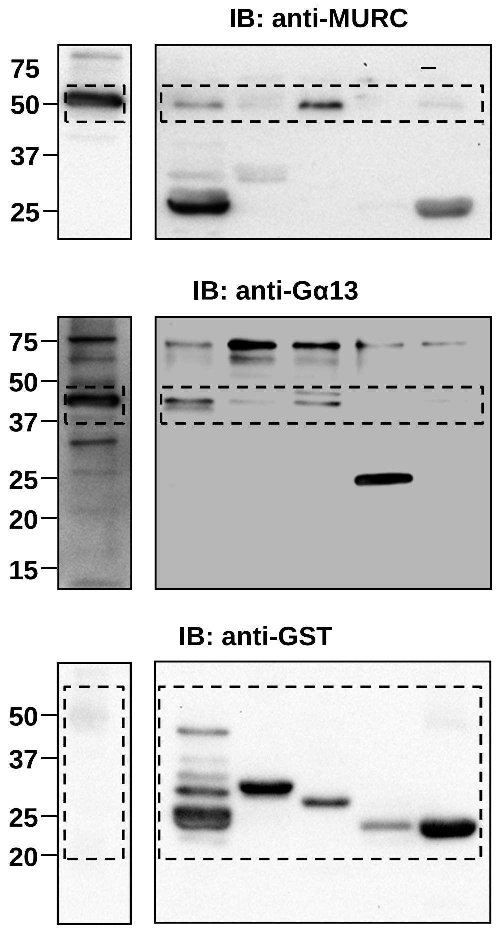

Fig. 7b

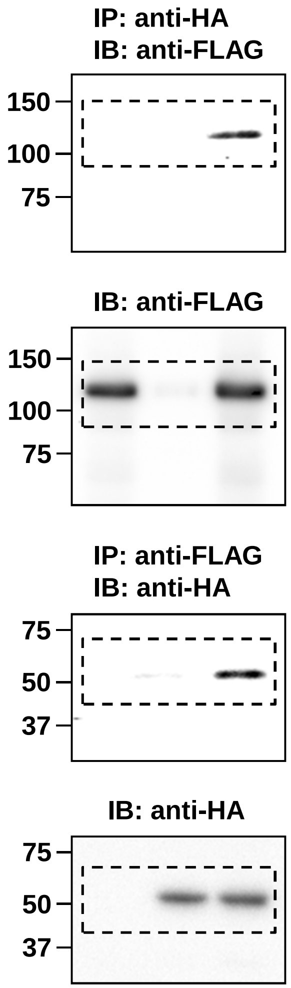

Fig. 7d

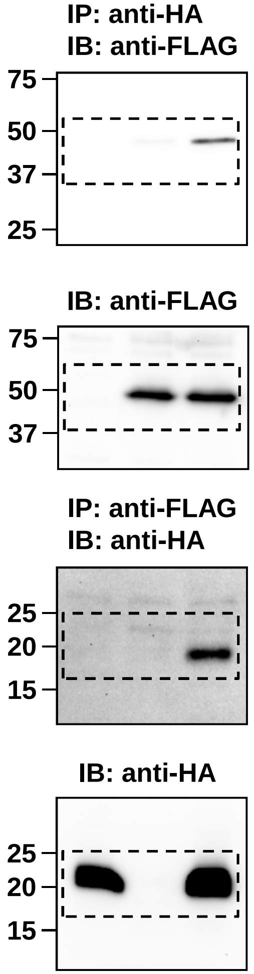

Fig. S1a

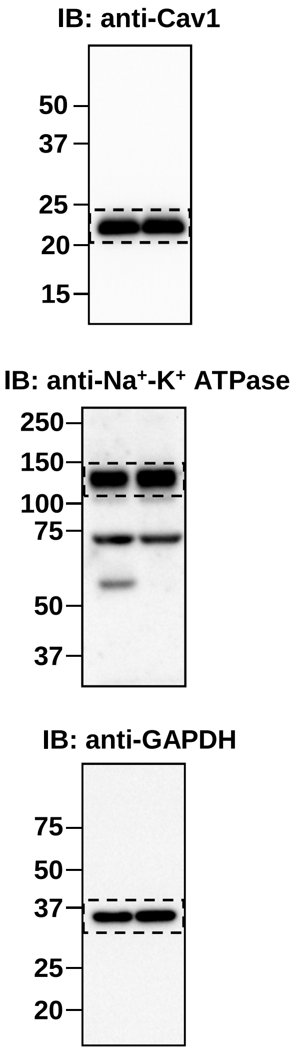

Fig. S1b

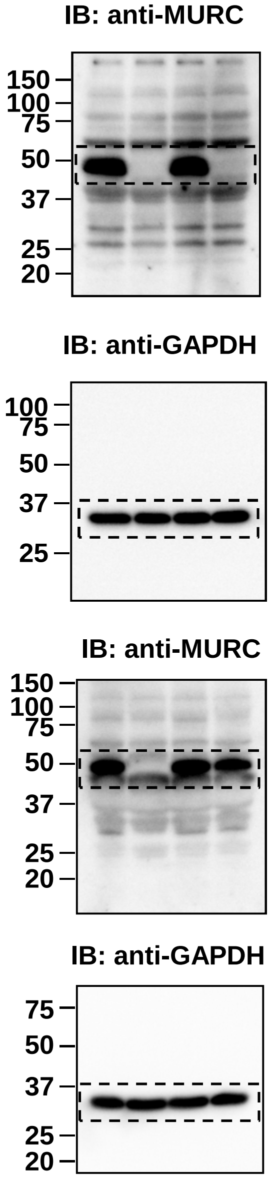

Fig. S3a

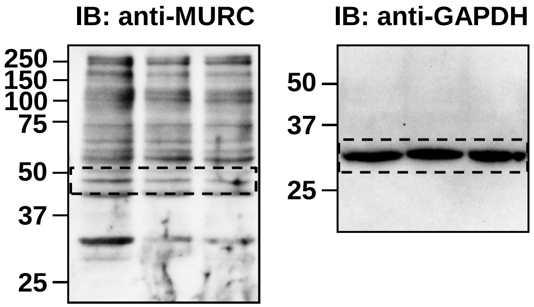

Fig. S3c

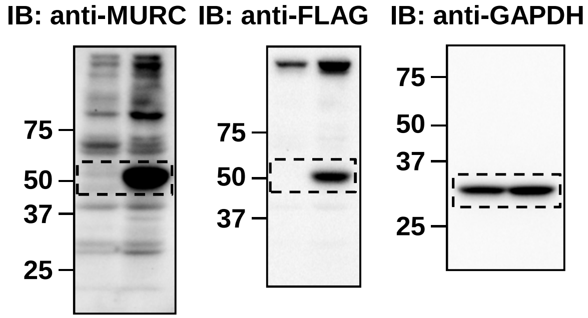

Fig. S3g

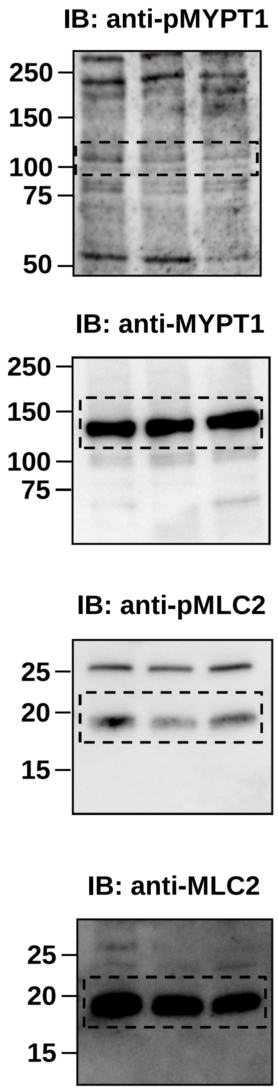

Fig. S3h

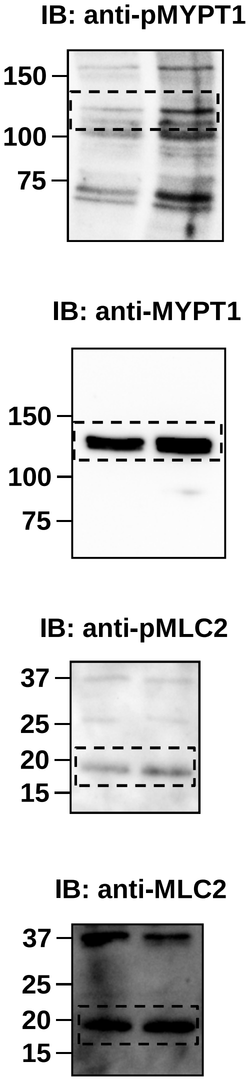

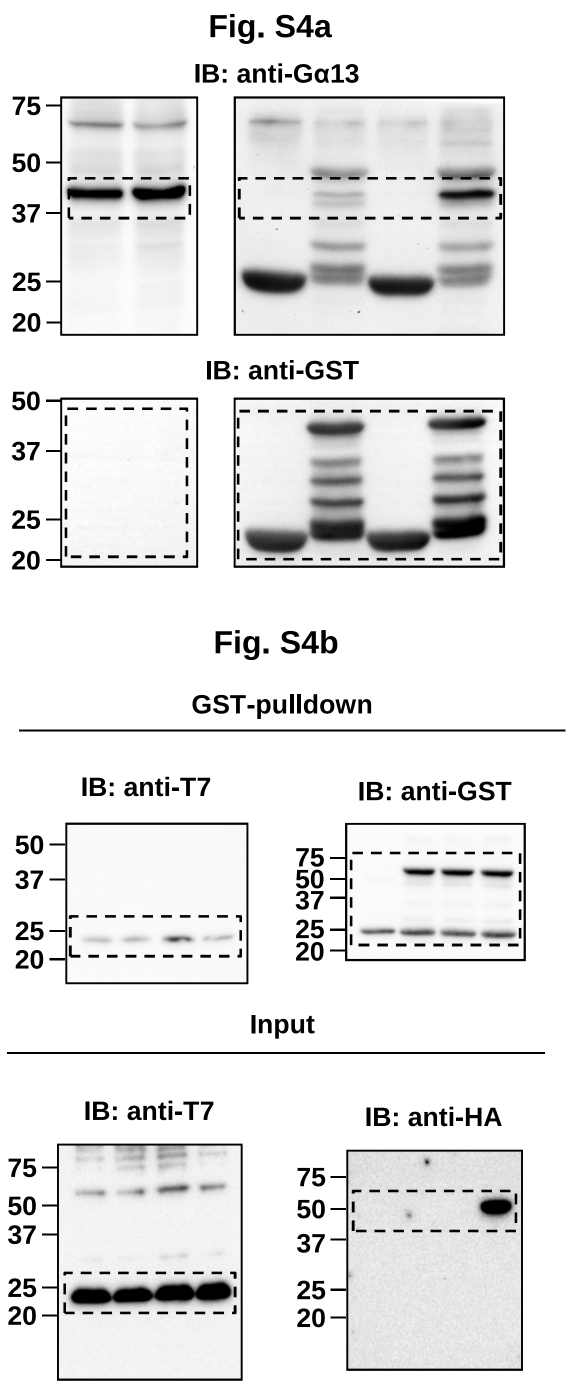

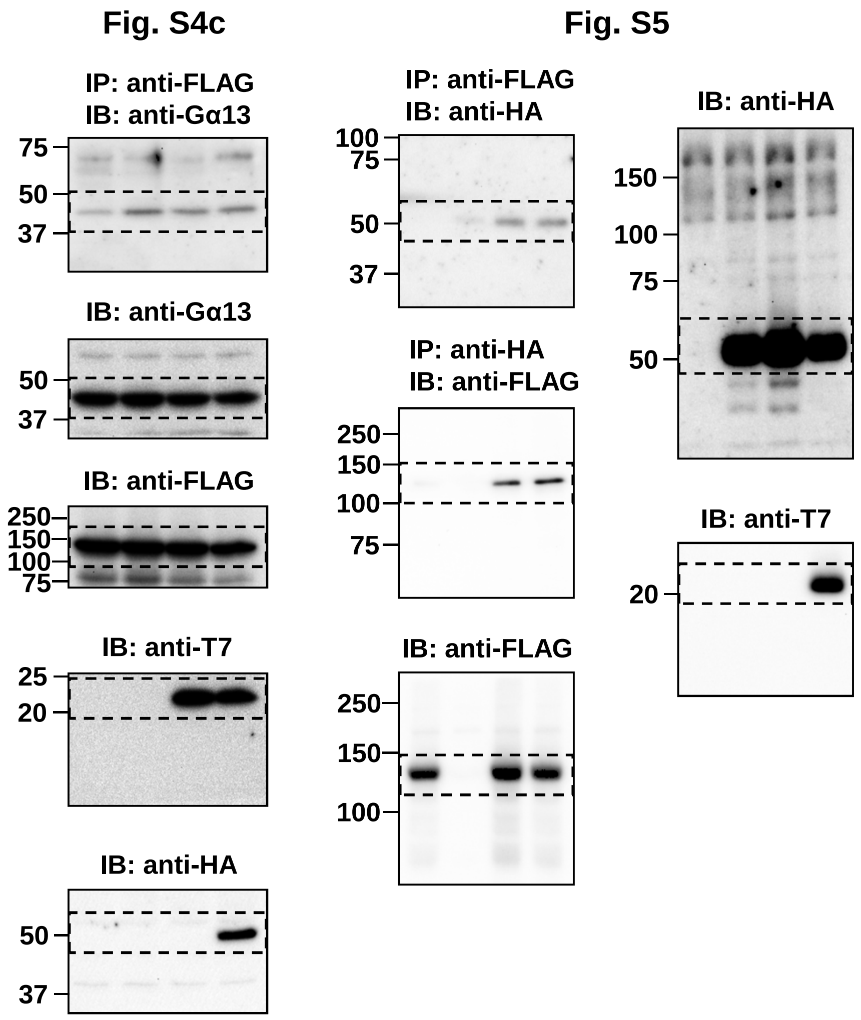

Supplementary Figure 6. Uncropped blots in main and supplementary figures.

Full immunoblot images with the corresponding figure and panel numbers are shown.

Supplementary Table 1. Blood pressure, heart rate, and echocardiographic analyses of WT and *Murc*$^{-/-}$ mice under normoxia

	WT (n=10)	*Murc*$^{-/-}$ (n=11)
sBP (mmHg)	93.5 ± 1.2	94.1 ± 1.3
dBP (mmHg)	62.8 ± 1.7	62.0 ± 1.6
HR (bpm)	676.0 ± 9.3	665.5 ± 16.5
LVDd (mm)	4.41 ± 0.30	4.07 ± 0.06
LVDs (mm)	2.90 ± 0.04	2.84 ± 0.03
IVSTd (mm)	0.60 ± 0.03	0.53 ± 0.02
PWTd (mm)	0.59 ± 0.18	0.56 ± 0.01
FS (%)	29.3 ± 0.4	30.1 ± 0.7
EF (%)	56.7 ± 0.7	57.8 ± 0.9

sBP, systolic blood pressure; dBP, diastolic blood pressure; HR, heart rate; LVDd, left ventricular dimension at end-diastole; LVDs, left ventricular dimension in systole; IVSTd, interventricular septum thickness at end-diastole; PWTd, left ventricular posterior wall thickness at end-diastole; FS, fractional shortening; EF, ejection fraction. Values are expressed as means ± SEM.

Supplementary Table 2. Morphometric and echocardiographic analyses of *Murc*$^{fl/fl}$ and cKO mice under normoxia

	Murc$^{fl/fl}$ (n=3)	cKO (n=3)
BW (g)	29.2±0.3	27.6±0.8
HW (mg)	120.6±1.8	122.7±3.3
TL (mm)	17.2±0.17	16.7±0.19
HW/BW (mg/g)	4.13±0.02	4.44±0.01
HW/TL (mg/mm)	7.01±0.04	7.34±0.12
LVDd (mm)	3.81±0.03	3.79±0.13
LVDs (mm)	2.65±0.03	2.56±0.05
IVSTd (mm)	0.62±0.02	0.64±0.02
PWTd (mm)	0.61±0.01	0.63±0.03
FS (%)	30.4±1.1	32.3±2.1
EF (%)	58.6±1.6	61.2±2.8

BW, body weight; HW, heart weight; TL, tibial length; LVDd, left ventricular dimension at end-diastole; LVDs, left ventricular dimension in systole; IVSTd, interventricular septum thickness at end-diastole; PWTd, left ventricular posterior wall thickness at end-diastole; FS, fractional shortening; EF, ejection fraction. Values are expressed as means ± SEM.

Supplementary Table 3. Morphometric and echocardiographic analyses of *Murc*$^{fl/fl}$ and cKO mice exposed to hypoxia

	Murc$^{fl/fl}$ (n=3)	cKO (n=3)
BW (g)	21.9±1.5	24.0±0.8
HW (mg)	111.4± 4.0	128.2±2.6
TL (mm)	17.7±0.18	18.3±0.15
HW/BW (mg/g)	5.11±0.26	5.36±0.12
HW/TL (mg/mm)	6.30±0.16	7.01±0.09
LVDd (mm)	3.41±0.22	3.51±0.11
LVDs (mm)	2.26±0.13	2.37±0.12
IVSTd (mm)	0.58±0.02	0.59±0.02
PWTd (mm)	0.59±0.01	0.61±0.02
FS (%)	34.0±1.0	32.6±1.4
EF (%)	64.1±1.3	61.9±2.0

BW, body weight; HW, heart weight; TL, tibial length; LVDd, left ventricular dimension at end-diastole; LVDs, left ventricular dimension in systole; IVSTd, interventricular septum thickness at end-diastole; PWTd, left ventricular posterior wall thickness at end-diastole; FS, fractional shortening; EF, ejection fraction. Values are expressed as means ± SEM.
